# Unbiased analysis of senescence associated secretory phenotype (SASP) to identify common components following different genotoxic stresses

**DOI:** 10.18632/aging.100971

**Published:** 2016-06-09

**Authors:** Servet Özcan, Nicola Alessio, Mustafa B. Acar, Eda Mert, Fatih Omerli, Gianfranco Peluso, Umberto Galderisi

**Affiliations:** ^1^ Genome and Stem Cell Center (GENKOK), Erciyes University, Kayseri, Turkey; ^2^ Department of Biology, Faculty of Sciences, Erciyes University, Kayseri, Turkey; ^3^ Sbarro Institute for Cancer Research and Molecular Medicine, Center for Biotechnology, Temple University, Philadelphia, PA 19122, USA; ^4^ Department of Experimental Medicine, Biotechnology and Molecular Biology Section, Second University of Naples, Naples, Italy; ^5^ Institute of Bioscience and Bioresources, CNR, Naples, Italy

**Keywords:** mesenchymal stem cells, senescence, secretome

## Abstract

Senescent cells secrete senescence-associated secretory phenotype (SASP) proteins to carry out several functions, such as sensitizing surrounding cells to senesce; immunomodulation; impairing or fostering cancer growth; and promoting tissue development. Identifying secreted factors that achieve such tasks is a challenging issue since the profile of secreted proteins depends on genotoxic stress and cell type. Currently, researchers are trying to identify common markers for SASP. The present investigation compared the secretome composition of five different senescent phenotypes in two different cell types: bone marrow and adipose mesenchymal stromal cells (MSC). We induced MSC senescence by oxidative stress, doxorubicin treatment, X-ray irradiation, and replicative exhaustion. We took advantage of LC-MS/MS proteome identification and subsequent gene ontology (GO) evaluation to perform an unbiased analysis (hypothesis free manner) of senescent secretomes. GO analysis allowed us to distribute SASP components into four classes: extracellular matrix/cytoskeleton/cell junctions; metabolic processes; ox-redox factors; and regulators of gene expression.

We used Ingenuity Pathway Analysis (IPA) to determine common pathways among the different senescent phenotypes. This investigation, along with identification of eleven proteins that were exclusively expressed in all the analyzed senescent phenotypes, permitted the identification of three key signaling paths: MMP2 - TIMP2; IGFBP3 - PAI-1; and Peroxiredoxin 6 - ERP46 - PARK7 - Cathepsin D - Major vault protein. We suggest that these paths could be involved in the paracrine circuit that induces senescence in neighboring cells and may confer apoptosis resistance to senescent cells.

## INTRODUCTION

Several genomic stressor events, such as telomere shortening, non-telomeric DNA damage, extreme mitogenic signals, and alteration of chromatin organization, may induce cellular senescence, which arrests cell division and induces loss of cell's functions. Senescence can counteract cancer growth since it blocks the proliferation of transformed cells, but it also contributes to organismal aging. Recently, evidence has emerged that senescence is involved in other biological processes such as tissue repair and development [1, 2].

Senescent cells present several attributes including flattened and enlarged morphology; increase in β-galactosidase activity; telomere-dysfunction-induced foci; senescence-associated heterochromatin foci; DNA scars (DNA segments with chromatin alterations reinforcing senescence); altered gene expression; and a senescence-associated secretory phenotype (SASP).

Collectively, this SASP accomplishes several tasks. It can sensitize surrounding cells to senesce to avoid situations where normal neighboring cells, some of them with minimal DNA damage, fail to enter senescence. SASP can attract and activate immune system cells to dispose senescent cells. SASP also produces detrimental physiological consequences. It can undermine tissue and organ functionality and contribute to organismal aging. Moreover, tumor cells can misuse SASP for their own growth. Actually, the inflammatory cytokines, growth factors, and proteases present in SASP allow the modification of tissue micro-environment that support tumor growth by promoting angiogenesis, epithelial–mesenchymal transition, and cancer cell proliferation [2-6]. All these phenomena appear related to cancer priming of senescent cells, i.e. contact between cancer cells and senescent cells heavily modify SASP composition [7].

In the current research, we decided to study the SASP produced by bone marrow and adipose mesenchymal stromal cells (MSC), which contains a subpopulation of stem cells able to differentiate in mesodermal derivatives (e.g., adipocytes, chondrocytes, osteocytes) and can also contribute to the homeostatic maintenance of several organs [8]. Furthermore, MSCs are under scrutiny in numerous clinical trials for the treatment several human diseases [9].

MSCs accomplish their functions through the secretion of cytokines and growth factors, which exert paracrine and autocrine functions [10]. Senescence greatly alters the composition of this secretome and hence impairs one of the key MSC biological functıons.

On these premises, the study of MSC SASP in different experimental conditions may illuminate the key functions exerted by senescent secretomes under different genotoxic stresses and the impairment of MSC autocrine and paracrine activity that may occur in senescence.

## RESULTS

Senescent phenotypes can be classified according to the stressor type into acute or chronic senescent cells.

Exogenous stressors may trigger acute senescence to arrest the damaged cell growth or become part of physiological phenomena (embryonic development and tissue repair). Continuous proliferation is associated with the DNA replication process and consequent accumulation of genomic damage. This enduring stress induces senescence called chronic senescence or, alternatively, replicative senescence [2].

**Figure 1:**
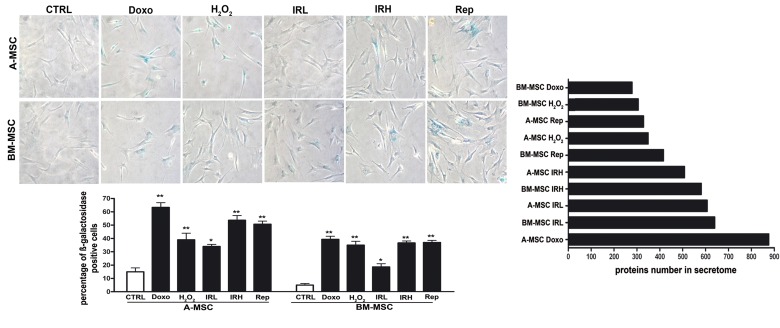
Induction of senescence in MSC cultures Left: Representative microscopic fields of acid beta-galactosidase (blue) in treated and control cells are shown. The histogram shows mean percentage value of senescent cells (± SD, n = 3, *p < 0.05; p**<0.01). Right: The graph shows the number of proteins found in secretomes of senescent A MSC and BM MSC. DOXO and H2O2 indicate the doxorubicin- and peroxide-treated MSCs Low (40 mGy) and high (2000 mGy) dose irradiated cells were indicated as IRL and IRH, respectively. Replicative senescent MSCs were called REPs.

We induced senescence on bone marrow and adipose mesenchymal stromal cells (BM MSC and A MSC), which share several biological features but also demonstrate peculiarities in their differentiation potential, transcriptome, proteome, and immuno-modulation function [14]. In a preliminary step, we used acid beta-galactosidase assay to assess senescence levels following treatment with different stressors (oxidative stress, doxorubicin treatment, X-ray irradiation, and replicative exhaustion). We named DOXO and H2O2, the doxorubicin- and peroxide-treated MSCs. Cells treated with low (40 mGy) and high (2000 mGy) radiations were indicated as IRL and IRH, respectively. Replicative senescent MSCs were called REPs. In all our experimental conditions, the number of senescent cells increased significantly with at least a twofold increase in senescence levels, as shown in Figure 1.

To identify protein secretome composition, we conducted LC-MS/MS analyses on peptides from the tryptic digestion of secretome samples. Using high-resolution MS in a search of the Protein Metrics database, several hundred proteins were identified in all senescence conditions, ranging from 279 in BM MSC DOXO to 876 in A MSC DOXO, as shown in Figure 1, and Supplementary Files 1 and 2. As demonstrated in our previous research [7], a huge fraction of proteins present in the secretomes lacked the signal peptide for the classical endoplasmic reticulum–Golgi secretion pathway. Indeed, it is well known that cells export proteins and nucleic acids in biological fluids via extracellular vesicles (exosomes and microvesicles). This is a way for intercellular communication to transfer membrane-bound and cytosolic proteins, lipids, and RNA among cells.

### Gene ontology analysis with PANTHER online software

High-throughput experiments, such as proteome analysis, enable the interrogation of thousands of data points simultaneously. These data have to be systematically organized in order to obtain biological information.

Gene Ontology (GO) performs an enrichment analysis to evaluate the relative frequency of biological functions, named ontology terms, in the proteomic profile of interest. GO is based on a database of three non-overlapping controlled vocabularies that describe molecular functions, biological processes, and cellular components.

We used PANTHER gene ontology enrichment analysis to match our experimental data onto reference ontology terms. This allowed the identification of ontology terms that occurred more frequently in our datasets when compared with a reference protein set. This experimental approach enabled the identification of protein sets of interest in a hypothesis free manner [15, 16].

We focused our GO analysis looking for two ontological terms: biological functions and molecular processes. For each of the ten different experimental conditions, we identified dozens of ontologies, as shown in Supplementary File 3. We used a Venn diagram to combine the data of all experimental conditions in order to find the biological processes and the molecular functions shared among the different senescent phenotypes, as shown in Table 1. In the senescent BM MSC phenotypes, we found 15 common ontologies belonging to biological processes. The same occurred for senescent A MSC cells. Of these, 13 were shared by the two cells types, as shown in Figure 1. BM MSC and A MSC senescent cells showed 15 and 13 common molecular function ontologies, respectively. All the common ontologies of A MSC were also present in BM MSC senescent cells, as shown in Figure 1. GO analysis allowed a preliminary identification of the most meaningful components of senescent secretomes. Indeed, the common ontologies we identified can be grouped into four classes: i) Extracellular matrix/cytoskeleton/cell junctions; ii) metabolic processes; iii) ox-redox factors; and iv) regulators of gene expression.

**Table 1:** Gene Ontology (GO) analysis

	Biological process		Molecular function	
**Extracellular matrix/cytoskeleton/cell junctions**	cellular component organization cellular component morphogenesis cellular component organization or biogenesis	(GO:0016043) (GO:0032989) (GO:0071840)	cytoskeletal protein binding structural molecule activity actin binding structural constituent of cytoskeleton	(GO:0008092) (GO:0005198) (GO:0003779) (GO:0005200)
**metabolic processes**	regulation of nucleobase-containing compound metabolic process glycolysis generation of precursor metabolites and energy RNA metabolic process protein metabolic process tricarboxylic acid cycle	(GO:0019219) (GO:0006096) (GO:0006091) (GO:0016070) (GO:0019538) (GO:0006099)		
**ox-redox factors**			oxidoreductase activity antioxidant activity peroxidase activity	(GO:0016491) (GO:0016209) (GO:0004601)
**regulators of gene expression**	transcription from RNA polymerase II promoter regulation of transcription from RNA polymerase II promoter protein folding transcription, DNA-dependent	(GO:0006366) (GO:0006357) (GO:0006457) (GO:0006351)	sequence-specific DNA binding transcription factor activity nucleic acid binding transcription factor activity	(GO:0003700) (GO:0001071)
**miscellaneous**			isomerase activity peptidase activity catalytic activity peptidase inhibitor activity	(GO:0016853) (GO:0008233) (GO:0003824) (GO:0030414)

The table shows the ontologies that were common to the senescent secretomes. Ontologies belong to “biological process” and “molecular function” categories. The identified ontologies were divided into four groups plus a miscellaneous collection.

### Identification of senescent-specific canonical pathways

GO scrutiny is useful for an overall description of the key functionalities in the samples under analysis, but the enriched ontology terms, found in experimental datasets, cannot directly pinpoint the most important proteins in the analyzed proteomes.

In order to reach a deeper insight on the most meaningful proteins in proteome samples, we used IPA to identify the set of interactions for each protein in our datasets and compared this with a reference interactome dataset [15]. The key assumption is that the connectivity of a protein found in senescent secretomes reflects its functional importance for the phenotype. Under this premise, we conducted an IPA canonical pathway analysis to evaluate how proteins present in senescent secretomes could be attributed to well established and classically characterized pathways.

**Figure 2:**
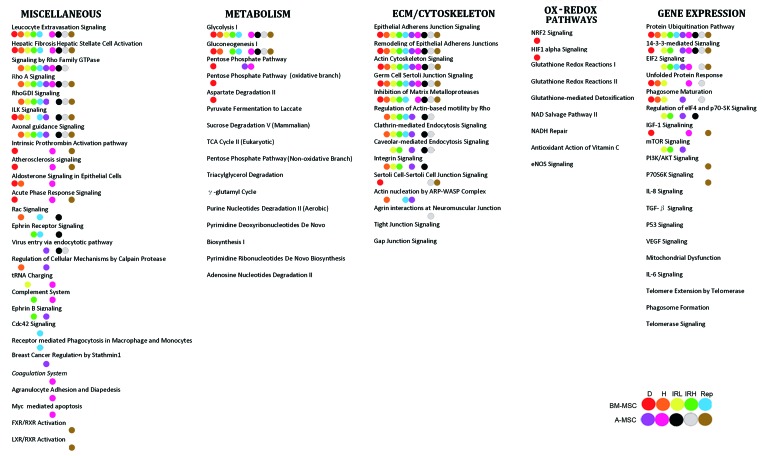
Common canonical pathways The figure shows the pathways common to senescent secretomes. Pathways were grouped according to the classes we identified in the GO analysis. For every experimental condition indicated in the figure with colored dots, IPA analysis allowed the identification of paths that belonged to overlapping networks.

For every senescent phenotype, the proteins present in the secretome could be attributed to hundreds of canonical pathways, as shown in Supplementary File 4. We used a Venn diagram to combine data of all experimental conditions in order to find the common canonical pathways among the different senescent phenotypes, as shown in Supplementary File 5. In this way, we identified 138 canonical pathways present in all the tested experimental conditions. Most of the common canonical pathways could be grouped according the four classes identified in the GO analysis: extracellular matrix/cytoskeleton/cell junctions; metabolic processes; ox-redox factors; and regulators of gene expression (Figure 2). Subsequently, we identified the overlapping networks present in any experimental condition to further dissect the most meaningful canonical pathways among the common 138 senescent secretome pathways. Overlapping networks are assembled based on the consideration that a given protein may be a part of different pathways, based on the assumption that the more a protein is present in overlapping pathways, the more influence it has on the senescence phenotype. For the extracellular matrix class, the common overlapping networks were epithelial adherens junction signaling and remodeling; inhibition of matrix metalloproteases; germ cell sertoli junction; and hepatic fibrosis. This suggests that modification of the extracellular environment is one of the main tasks of the senescence secretome. In the gene regulation class we found a “protein ubiquitination pathway.” Finally, we identified “leucocyte extravasation signaling” as an overlapping network that is common among the different senescence secretomes. This is important since SASP contains several pro-inflammatory cytokines and inflammation phenomena are associated with the movement of leukocytes out of the circulatory system (extravasation) and towards the site of tissue damage or infection. A complete list of canonical pathways that were in overlapping networks in all of the senescent phenotypes is shown in Table 2.

**Table 2:** Overlapping networks

	A MSC	BM MSC	COMMON
**INHIBITION OF MMP**	A2M, MMP2, TIMP1, TIMP2	A2M, MMP2, TIMP2	A2M, MMP2, TIMP2
**EPITHELIAL ADHERENS JUNCTION**	ACTN1, ACTN4, ACTR2, MYH9, TUBA1C, TUBB, VCL	ACTN1, ACTN4, ACTR2, ARPC3, IQGAP1, MYH9, TUBB, VCL	ACTN1, ACTN4, ACTR2, MYH9, TUBB, VCL
**REMODELING OF EPITHELIAL ADHERENS JUNCTION**	ACTN1, ACTN4, ACTR2, TUBA1C, TUBB, VCL	ACTN1, ACTN4, ACTR2, IQGAP1, NME1, TUBB, VCL	ACTNI, ACTN4, ACTR2, TUBB, VCL
**GERM CELL SERTOLI JUNCTION**	A2M, ACTN1, ACTN4, TUB1AC, TUBB, VCL	A2M, ACTN1, ACTN4, CFL1, IQGAP1, TUBB, VCL	A2M, ACTN1, ACTN4, TUBB, VCL
**ACTIN CYTOSKELETON**	ACTN1, ACTN4, ACTR2, EZR, MSN, MYH9, PFN1, TLN1, VCL	ACTN1, ACTN4, ARPC3, CFL1, FN1, IQGAP1, MSN, PFN1, RDX, VCL	ACTN1, ACTN4, MSN, PFN1, VCL
**HEPATIC FIBROSIS**	A2M, COL1A1, COL1A2, COL3A1, COL6A2, FN1, IGFBP3, MMP2, MYH9, SERPINE1, TIMP1, TIMP2	A2M, COL1A1, COL1A2, COL3A1, FN1, IGFBP3, MMP2, MYH9, SERPINE1, TIMP2	A2M, COL1A1, COL1A2, COL3A1, FN1, IGFBP3, MMP2, MYH9, SERPINE1, TIMP2
**PROTEIN UBIQUITINATION**	HSP90AA1, HSP90AB1, HSP90B1, HSPA5, HSPA8, HSPB1, HSPD1, PSMA1, PSMA3, PSMA5, PSMA6	HSP90AA1, HSP90AB1, HSP90B1, HSPA4, HSPA5, HSPA8, HSPB1, HSPD1, PSMA5, PSMB1, PSMB4, PSMB5, PSMB6	HSP90AA1, HSP90AB1, HSP90B1, HSPA8, HSPD1, PSMA5
**RHOA SIGNALING**	ACTR2, EZR, MSN, PFN1	ACRT2, ARPC3, CFL1, MSN, PFN1	ACTR2, MSN, PFN1
**LEUCOCYTE EXTRAVASATION**	ACTN1, ACTN4, EZR, GNAI2, MMP2, MSN, TIMP1, TIMP2, VCL	ACTN1, ACTN4, MMP2, MSN, RDX, TIMP2, VCL	ACTN1, ACTN4, MMP2, MSN, TIMP2, VCL

The 138 canonical pathways that were common among the different senescent secretomes were further analyzed to find the overlapping networks. The table shows the canonical pathways that were in overlapping networks in the great majority (9/10) of senescent phenotypes.

For every canonical pathway (rows), this table shows the proteins present in the senescent secretomes of BM MSC, A MSC, and those common to the two cell types.

A closer look at the list of the proteins belonging to overlapping canonical pathways evidenced the presence of several structural proteins (ACTN1, ACTN4, TUBB, VCL, MSN, PFN1); extracellular remodelers (COL1A1, COL1A2, COL3A1, MMP2, TIMP2); and proteins of misfolding and degradation signaling (HSP90AA1, HSP90AB1, HSP90B1, HSPA8, HSPD1).

### Identification of senescent secretome specific proteins

We then focused on the identification of proteins exclusively expressed in all the SASPs and absent in the secretomes of healthy functional MSCs. We collected the conditional media from early passage BM MSC and MSC, then performed the LC-MS/MS analyses on peptides from the tryptic digestion of secretome samples. Using high-resolution MS in a search of the Protein Metrics database, we found 372 proteins in early passage BM MSC and 254 in A MSC, as shown in Supplementary File 2. Venn analysis allowed us to identify 11 proteins that were exclusively expressed in all the analyzed senescent phenotypes, as shown in Table 3. Of interest, nine of these proteins belonged to the classes we identified with the GO analysis. An in depth analysis of these proteins and of those belonging to overlapping canonical pathways allowed us to hypothesize, as reported in the discussion, that some could be involved in key functions of senescent cells.

**Table 3:** Proteins exclusively expressed in the senescent phenotypes

	Name of Protein	UniProt ID
**Extracellular matrix/cytoskeleton/cell junctions**	Filamin B Tubulin alpha 1C chain Tubulin beta chain	E7EN95 F5H5D3 Q5JP53
**metabolic processes**		
**ox-redox factors**	Peroxiredoxin 6 Protein deglycase DJ-1 (PARK7) TXNDC5 (ERP46)	P30041 Q99497 I3L3M7
**regulators of gene expression**	Major vault protein 14-3-3 protein epsilon Proteasome subunit beta type 4	Q14764 P62258 P28070
**miscellaneous**	Aminopeptidase N (CD13) Cathepsin D	P15144 P07339

Venn analysis allowed the identification of 11 proteins that were exclusively expressed in all the senescent phenotypes and absent in the secretomes of healthy functional MSCs. The proteins were divided into GO groups plus a miscellaneous collection.

## DISCUSSION

Currently, researchers are trying to identify the common features for SASPs. The present investigation compared the secretome composition of five different senescent phenotypes in two different cell types (BM MSC and A MSC). These cells were selected because their activities are accomplished by producing factors acting in both paracrine and autocrine signaling processes. Changes in the secretome profile as it passes from a healthy to a senescent state greatly affects the MSC physiology.

Many researchers have focused their analysis of senescent secretomes on specific proteins, such as the released factors that may modify extracellular environment (proteases, ECM components), proteins that promote inflammation (interleukins, chemokines), as well as those that affect the behavior of cancer cells (growth factors) [3]. We took advantage of LC-MS/MS proteome identification and subsequent gene ontology evaluation to perform an unbiased analysis (hypothesis free manner) of senescent secretomes. We compared SASPs following chemical and physical stresses and following replicative exhaustion.

Our results are in good agreement with the hypothesis that senescent program includes two distinct events: cell cycle arrest and geroconversion. Cells may arrest and preserve their ability to re-enter cell cycle o, alternatively, they may undergo geroconversion. This latter phenomenon leads to permanent cell cycle arrest, hypertrophy and hypersecretion (SASP) [17, 18].

### Identification of new SASP categories

GO analysis allowed us to distribute SASP components across four classes: extracellular matrix/cytoskeleton/cell junctions; metabolic processes; ox-redox factors; and regulators of gene expression. These classes increase the number of SASP categories that have been identified so far [3] and demonstrate the importance of secretome elements in regulation cellular oxidative status and metabolism. The oxidative hypothesis of senescence dated back to the 1950s, but it has not been able to completely explain the interplay between oxidative injuries and the complex network of intracellular redox regulatory system [1, 19, 20]. Our finding suggests that whatever the senescence inducer stimulus, once cells enter senescence, they release modulators of ox-redox status to affect neighbor cells. Is well known that changes in cellular status are associated with metabolic modifications and that blocking these metabolic switches may affect cellular status. For example, cancer cells rely mainly on anaerobic glycolysis; forcing the switch of their metabolism to ATP production through the TCA cycle may promote the loss of cancer cell features with a loss of tumorigenicity. In our previous work, we demonstrated that senescent cells have metabolic inflexibility and prefer to use glucose as energy fuel in the TCA cycle [21]. Current research suggests that modifications in metabolism may be important for triggering senescent phenotypes since the factors involved in metabolic processes are released by senescent cells to influence surrounding cells.

### Key circuits for paracrine activity of senescent cells

**Figure 3:**
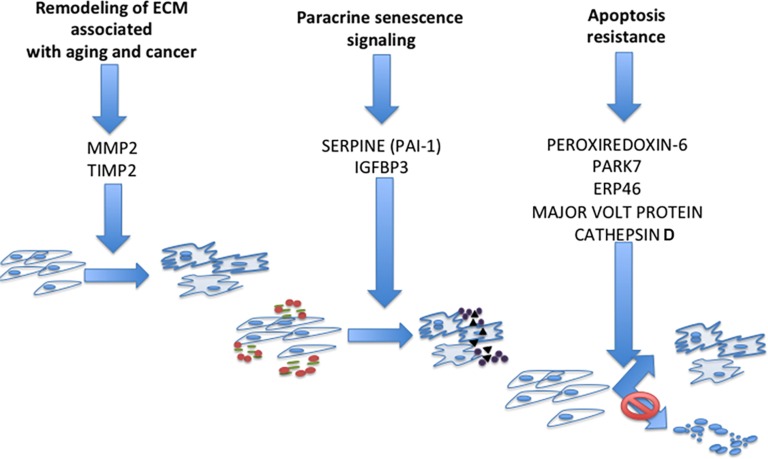
Circuits that mediate paracrine interactions between senescent cells and surrounding environment According to experimental results and literature data we propose: MMP2 and TIMP2 may play a key role in pathways regulating the effect of senescent secretomes on cancer cells; IGFBP3 and PAI-1 are part of the paracrine circuit that induces senescence in neighboring cells; and PRDX6, ERP46, PARK7, Cathepsin D, and MVP could be part of the common circuit that allows senescent cell survival following stress.

We used IPA analysis to identify common canonical pathways among the different senescent phenotypes. The list of the proteins belonging to overlapping canonical pathways demonstrated the presence of several structural proteins, extracellular remodelers, and proteins of misfolding and degradation signaling. The examination of these proteins, along with the 11 proteins that were exclusively expressed in all the analyzed senescent phenotypes, allowed us to hypothesize that these factors are part of some key circuits that mediate paracrine interactions between senescent cells and surrounding environment. As shown in Figure 3, we identified the following paths: MMP2 - TIMP2; IGFBP3 – PAI-1 (SERPINE1); and Peroxiredoxin 6 – PARK7 – ERP46 – Major vault protein – Cathepsin D.

Matrix metalloproteinases (MMPs) play a key role in physiological and pathological remodeling of the ECM during embryogenesis, angiogenesis, wound healing, metastasis, and inflammatory diseases [22-24]. The tissue inhibitors of metalloproteinases (TIMPs) regulate the proteolytic activity of MMPs by complexing with and inactivating them [25]. In the secretome of senescent cells, several MMPs and TIMPs were detected depending on cell type or stress inducer. We found MMP2 and TIMP2 in all the common overlapping canonical pathways, suggesting a preeminent role of these two proteins compared to the other MMPs and TIMPs.

MMP2 is found in most tissues and cells, and besides its activity as an ECM degrading enzyme, it also acts on several nonmatrix substrates, such as stromal cell-derived factor 1, big endothelin-1, interleukin 1β precursor, and monocyte chemoattractant protein-3. This suggests that MMP2 plays a key role in promoting and auto-regulating the inflammation process [26-28]. TIMP2, besides its activity in regulating MMPs, including MMP2, has a role in negatively regulating cellular response to growth factors, thus arresting cancer sprouting. On the other hand, it can also impair physiological homeostasis as it can occur in tissues containing senescent cells [29-32]. The MMP2-TIMP2 circuit may contribute to the apparently contradictory functions of SASP: promotion and arrest of cancer growth.

Factors regulating the IGF signaling pathways are expressed in senescent secretomes [3]. Our previous research detected several members of the Insulin like growth factors binding proteins (IGFBPs) in the secretome of senescent MSC and found a causative role for IGFBP4 and IGFBP7 [33]. Other findings reported a role in senescence for IGFBP3 [34, 35]. Indeed, we found expressed IGFBP3 and PAI-1 (SERPINE1) in all the common overlapping canonical pathways. Cells incubated in media containing IGFBP3 enter senescence, but this is blocked by tissue plasminogen (t-PA), which degrades IGFBP3. PAI-1 prevents IGFBP3 proteolysis by t-PA and induces senescence [34]. These reports suggest that senescent cells may secrete IGFBP3 and its natural “safeguard” molecule, PAI-1, as part of the paracrine circuit that induces senescence in neighboring cells. In support of this hypothesis, Eren and collaborators found that PAI-1 deficiency in mice retards the development of senescence, protecting organs structure and function [36].

Reactive Oxygen Species (ROS) play a central role as mediators of senescence regardless of the genotoxic stress that induces senescence. ROS concentration has to be strictly controlled to avoid a huge increase in oxidative phenomenatriggering apoptosis. It is well known that senescent cells are resistant to apoptosis, but there is no clear indication how this is accomplished [1]. Our findings may provide some hints. Peroxiredoxin 6 (PRDX6) is a non-seleno peroxidase, whose expression is induced following hyperoxia exposure. Similar to other peroxiredoxins, PRDX6 reduces peroxide hydrogen. It also has the ability to reduce peroxidized membrane phospholipids. Initiation of lipid peroxidation can produce progressive damage to cell plasma and other organelle membranes that results in apoptosis [37]. PRDX6 may protect cells under stress (such as senescent cells) from cell death. ERP46, also known as thioredoxin domain-containing 5, is a resident endoplasmic reticulum protein with a thioredoxin peroxidase activity. It can restore the oxidoreductase capacity of peroxiredoxins by electron transfer. Its decreased activity seems to be implicated in cell senescence [38]. PARK7 is a protein deglycase that repairs protein function by deglycating cysteine, arginine, and lysine residues. PARK7 can promote cell survival, protecting cells from oxidative stress by quenching ROS and conferring apoptosis resistance [39].

Cathepsin D is an acid protease active in intracellular protein breakdown. Several findings demonstrated that this protein participates in signaling that regulates cell death. Indeed, Byun and colleagues found that the Cathepsin D level may play part of the process governing cell decision about apoptosis and senescence following DNA damage stimulus [40]. They found that Cathepsin D was significantly overexpressed during senescence and decreased during apoptosis. Regulation of its expression may be part of cell strategy to regulate apoptosis and senescence pathways.

Major vault protein (MVP) is the main component of large ribonucleoparticles called vaults. The exact function of vaults and their components is still unknown. There are reports showing that the MVP gene is p53 responsive and the corresponding protein increases with age *in vivo* and *in vitro*. MVP upregulation facilitates apoptosis resistance of senescent cells [41-43].

We propose that PRDX6, ERP46, PARK7, Cathepsin D, and MVP could be part of the common circuit that allows senescent cell survival following stress. We also speculate that the presence of these proteins (singularly or in combination) may be part of the mechanism that regulates cell response to stress: either senescence or apoptosis.

## MATERIALS AND METHODS

### MSC cultures

Bone marrow was obtained from three male healthy donors (range: 6-10 years old) who provided informed consent. We separated cells on a Ficoll density gradient (GE Healthcare, Italy), and the mononuclear cell fraction was collected and washed in PBS. We seeded 1-2.5 × 10^5^ cells/cm^2^ in alpha-MEM containing 10% FBS and bFGF. After 72 hours, non-adherent cells were discarded and adherent cells were cultivated to confluency. Cells were then further propagated for the assays reported below.

Lipoaspirates were obtained from three male healthy donors (range 20 - 30 years old) undergoing plastic surgery after they provided their informed consent. The dispersion of adipose tissue was achieved via collagenase digestion, after which the lipid-filled adipocytes' ability to float caused them to separate from the stromal vascular fraction by way of centrifugation. Stromal pellets were washed with phosphate-buffered saline (PBS) and further purified on a density gradient (Histopaque, GE Healthcare, UK). Mononuclear cells fractions were collected and cultivated in Dulbecco's modified Eagle's medium containing 10% fetal bovine serum. These cells were further amplified to conduct experiments.

### Acute and chronic senescence

For the induction of acute senescence, we used three different stressors: irradiation, doxorubicin, and peroxide hydrogen treatments. Chronic senescent MSCs were obtained by extensive *in vitro* cultivation for 30 days (replicative senescence) as previously described [11].

### Irradiation treatment

Exponentially growing cells (passage 3) were irradiated with 40 and 2000 mGy X-rays at room temperature. X-rays were administered via a Mevatron machine (Siemens Italy) operating at 6 MeV. Following irradiation, cells were cultivated for 48 hours before carrying out further experiments.

### Doxorubicin treatment

Cells were incubated with 1 μM doxorubicin in complete culture medium for 24 hours, then the medium was discarded, and the cells were incubated for 24 hours in a fresh medium before further analysis.

### Peroxide hydrogen treatment

Cells were incubated with 300 μM H_2_O_2_ for 30 minutes in complete medium, then the medium was discarded, and the cells were incubated for 48 hours in a fresh medium before further analysis.

### In situ senescence-associated beta-galactosidase assay

The percentage of senescent cells was calculated by the number of blue beta-galactosidase-positive cells out of at least 500 cells in different microscope fields, as previously reported [12].

### CM preparation for LC-MS/MS analysis

Following genotoxic stress, we induced as reported in previous paragraphs, cells were incubated in serum free media for 24 hours to obtain conditioned media (secretomes). We did not observe increase in apoptosis after incubation in serum free media in all the experimental conditions.

Without disturbing the attached cells, 5 mL of MSC secretomes were collected from culture dishes and culture debris removed by centrifugation at 10,000 g. Supernatants were used for protein pooling with resin (StrataClean, Agilent Technology, CA, USA) using dried beads mixed with 1× Laemmli gel loading buffer and run on a gradient gel 4-15% SDS-PAGE (Criterion TGX Stain-Free Precast Gels, Bio-Rad, CA, USA). Following electrophoresis at 100 V, the gels were stained with Coomassie Brilliant Blue and gel lanes of interest were excised for in-gel digestion, as previously described [13].

After digestion, peptides were eluted from the gel matrix by immersing the reaction tube in an ultrasonic bath for 5 min with a sequential elution of 0.4% formic acid in 3% ACN, 0.4% formic acid in 50% ACN, and 100% ACN. The supernatant containing the peptides was centrifuged, transferred to low binding tubes, and desalted using pipette tips (ZipTip C18, Merck Millipore, Germany). Following that, the extracted peptides were dried and stored at −80°C until LC-MS/MS analysis was performed. A more detailed protocol of CM preparation appears in Supplementary File 2.

### LC-MS/MS analysis

Tandem mass spectrometric analysis was carried out using AB SCIEX TripleTOF 5600+ instrument (AB SCIEX, Redwood City, CA, USA) coupled to an Eksigent expert nano-LC 400 system (AB SCIEX). MS and MS/MS data was acquired using Analyst^®^ TF v.1.6 (AB SCIEX).

Mass spectrometry data was analyzed by using ProteinPilot 4.5 Beta (AB SCIEX) for the peptide identifications. The detailed protocol is described in Supplementary File 6.

### GO and network analyses

Proteins expressed in secretomes were analyzed with PANTHER (http://www.pantherdb.org) and Ingenuity Pathway Analysis (IPA) (http://www.ingenuity.com/products/ipa).

Using PANTHER, protein classification was performed according to three ontological terms: biological processes, molecular functions, and molecular classes. For PANTHER analysis, we used statistics overrepresentation (i.e., the default setting) to compare classifications of multiple clusters of lists with a reference list to statistically identify the over- or under-representation of PANTHER ontologies. Significance was set to a p-value of .05.

Differentially expressed proteins were imported into IPA to identify canonical pathways present in senescent secretomes. Fischer's exact test was used to calculate a p-value that would determine the probability that the association between genes in the dataset and canonical pathway could be explained by chance alone. Significance was set to a p-value of .05.

### Statistical analysis

Statistical significance was evaluated using ANOVA analysis followed by Student's t and Bonferroni's tests. We used mixed-model variance analysis for data with continuous outcomes. All data were analyzed with a GraphPad Prism version 5.01 statistical software package (GraphPad, CA, USA).

## CONCLUSION

We identified four classes of SASP components: extracellular matrix/cytoskeleton/cell junctions; metabolic processes; ox-redox factors; and regulators of gene expression. These classes are common among senescent phenotypes obtained with very different stressors, suggesting that they may represent a common feature of senescent secretome.

In addition, the 11 proteins we identified exclusively in all the analyzed senescent phenotypes need further investigations, since the fact that they can be found in very different experimental conditions suggests that they may represent a common signature of senescence phenomenon. Further investigations are needed to evaluate the role of the pathways we identified with our study. This could contribute to understand how senescence may be triggered by paracrine signaling pathways. The importance of these further investigations resides also on the consideration that senescence may affect the therapeutic performance of MSCs.

## SUPPLEMENTARY DATA AND TABLES














