# Supplementary material for: Unbiased analysis of senescence associated secretory phenotype (SASP) to identify common components following different genotoxic stresses
Source: Aging (Albany NY). 2016 Jun 9;8(7):1316–27. doi: 10.18632/aging.100971 (PMC4993333; doi:10.18632/aging.100971)
Supplement: Supplementary file 1 [file aging-08-1316-s001.pdf]

SUPPLEMENTARY DATA

Please browse the Full text version to see the Supplementary files of this manuscript.

Supplementary File 1
This file includes a list of proteins present in secretomes obtained from doxorubicin-, peroxide hydrogen-treated, low X ray treatment, high X ray treatment, and senescent replicative MSCs. For every experimental condition, there were at least two biological replicates.

Supplementary File 2
This file includes the Uniprot ID of proteins present in secretomes obtained from doxorubicin-, peroxide hydrogen-treated, low X ray treated, high X ray treated, and senescent replicative MSCs. The list also shows the proteins of healthy A MSC and BM MSC, indicated as AMSC early passage and BM MSC early passage, respectively.

Supplementary File 3
This file shows the GOslim analysis carried out with PANTHER database. The list shows ontology terms overrepresented in senescent secretomes. Ontology terms were classified in biological processes and molecular functions.

Supplementary File 4
This file lists the canonical pathways identified for every senescent secretome.

Supplementary File 5
This file lists the common canonical paths present in senescent cells.

Supplementary File 6. CM preparation for LC-MS/MS analysis

Binding of proteins on StartaClean beads

Five mL of naïve or ARH77-primed MSC secretomes were collected from culture dishes without disturbing the attached cells, then culture debris were removed by centrifugation 10000 g at 4°C. Supernatants of secretomes were either used for the StartaClean beads protein pooling or stored at -80 °C for further use. 20 µL of StrataClean beads (Agilent, USA) was added into 5 mL of each secretome sample, then incubated at 4°C overnight with gentle shaking or overhead rotation. The beads were precipitated by centrifugation at 8000 g at 4 °C for 15 min. Then supernatants were removed, the protein-loaded beads were washed two times with 500 µL of TE (50mM Tris, 10 mM EDTA, pH 8), transferred to a low binding microfuge tube and the beads were evaporated to dryness with a vacuum centrifuge.

Protein Elution with SDS-PAGE

Twenty µL of 1x Laemmli gel loading buffer supplemented with 20 mM DTT was added to dried affinity beads. The mixture slurry was boiled for 5 min, chilled on ice and total mixture loaded on a gradient gel 4-15% SDS-PAGE (Criterion TGX Stain Free Precast Gels, BIO-RAD, US). Electrophoresis was carried out at 100 V. The gel was pre-scanned (ChemiDocTM MP, BIO-RAD, US) then fixed with 10% (v/v) acetic acid in 40% (v/v) ethanol for 60 min and stained with colloidal Coomassie blue. After staining, the gel was rinsed twice with ddH2O to remove excessive Coomassie stain. The gel lanes of interest were excised. For each loading well, six to ten bands were excised and destained. In-gel digestion was carried out as described previously (Shevchenko, Tomas et al. 2006). After digestion, the peptides were eluted from the gel matrix by immersion of the reaction tube in an ultrasonic bath for 5 min with sequentially elution of 0.4% formic acid in 3% ACN, 0.4% formic acid in 50% ACN and 100% ACN. The supernatant containing the peptides was centrifuged, transferred to low binding tubes, desalted with ZipTip C18 (Millipore, Merck). Then, the extracted peptides were dried and stored at – 80°C until LC-MS/MS analysis.

LC-MS/MS analysis

Tandem mass spectrometric analysis was carried out using AB SCIEX TripleTOF 5600+ instrument (AB SCIEX, Redwood City, CA, USA) coupled to Eksigent expert nano-LC 400 system (AB SCIEX). Trap and elute mode was used to separate peptide mixture by LC system equipped with a trap column (180µm x 20mm column, 300 Å, nanoACQUITY UPLC® 2G-VM Trap 5µm Symetry® C18, Waters, UK) and a separation column (75µm, x 150mm column, nanoACQUITY UPLC® 1.8µm 120 Å HSS T3, C18, Waters, UK). Two µL of peptide samples were loaded onto the LC system with 10 µL loop valume in solvent A (0.1% formic acid (v/v) in H2O) for 10 min at a constant flow rate of 1µL/min with trapping. The peptide elution carried out using a linear gradient of 4-96% solvent B (0.1% formic acid in100% ACN) for 130 minutes at a constant flow rate of 250 nL/min. The gradient program was used as follows: the system was preconditioned with 96% solvent A (0.1% formic acid (v/v) in H2O) for 10 min. Following, solvent B (0.1% formic acid in 100% ACN) was increase from 4% to 50% with the duration of 95 min. Then, solvent B increased to 96% within 6 min; maintained for 7 min at 96% B / 4% A; then cleaning

for the next run was established. Elute was delivered into the mass spectrometer with a NanoSpray III source using a 10 μm ID nanospray emitter tip (New Objective, Woburn, MA). Electrospray ionization of floating voltage was maintained at 2400 V.

MS and MS/MS data was acquired using Analyst® TF v.1.6 (AB SCIEX). After a survey scans at a resolution of >35K in the m/z range of 350 to 1250 which exceeds 70 cps, high sensitivity on MS/MS mode with resolution in the m/z range of 100 to 1800 on both precursor and fragment of the 20 most abundant precursor ions were selected for fragmentation. A rolling Collision-induced dissociation (CID) fragmentation was performed for 25 ms with normalized collision energy of 10, declustering potential was set to 100 V and the fragment ions were recorded. Single and unassigned charge state precursor ions were not selected for MS/MS analysis. Mass tolerance was set to ±50 mDa. Mass spectrometer recalibration was performed at the start of each batch and repeated every third sample by using 25 fmol/μL β-galactosidase digest standard.

Mass spectrometry data analysis

Mass spectrometry data was analyzed by using ProteinPilot 4.5 Beta (AB SCIEX) for the peptide identifications. Following the extraction of all files, *.wiff files were used to carry out the peak list generation and database searches. In house human protein database was generated from UniProt Proteomes reference database of Homo sapiens (UP000005640, downloaded January 16, 2015). The parameters were used for database searches include trypsin as a protease with the allowance of one missed cleavage, and oxidation of methionine. Precursor ion mass error window of 10 ppm and fragment ion mass error window of 0.1 Da were allowed. The raw data obtained were searched against decoy database to calculate 1% false discovery (FDR). Proteins were only considered as identified if at least two unique peptides of which matching to a protein.

Shevchenko, A., H. Tomas, J. Havlis, J. V. Olsen and M. Mann. "In-gel digestion for mass spectrometric characterization of proteins and proteomes." Nat Protoc. 2006. 1: 2856-2860.
