# Supplementary material for: Unbiased analysis of senescence associated secretory phenotype (SASP) to identify common components following different genotoxic stresses
Source: Aging (Albany NY). 2016 Jun 9;8(7):1316–27. doi: 10.18632/aging.100971 (PMC4993333; doi:10.18632/aging.100971)
Supplement: Supplementary file 4 [file aging-08-1316-s004.pdf]

Now includes comprehensive GO annotations directly imported from the GO database

Analysis Summary: Please report in publication BM MSC REP BIOLOGICAL PROCESS

Analysis Type: PANTHER Overrepresentation Test (release 20150430)

Annotation Version and Release Date: PANTHER version 10.0 Released 2015-05-15

Analyzed List: Client Text Box Input (Homo sapiens) Change

Reference List: Homo sapiens (all genes in database) Change

Annotation Data Set: PANTHER GO-Slim Biological Process

☑ Use the Bonferroni correction for multiple testing

Results

	Reference list	Client Text Box Input
Mapped IDs:	20814	341
Unmapped IDs:	0	75

Export results View: -- Please select a chart to display --

Displaying only results with P<0.05; click here to display all results

	Homo sapiens (REF)	Client Text Box Input				
PANTHER GO-Slim Biological Process	#	#	expected	Fold Enrichment	+/-	P value
glycolysis (GO:0006096)	25	7	.41	> 5	+	5.61E-05
tricarboxylic acid cycle (GO:0006099)	20	5	.33	> 5	+	5.17E-03
regulation of liquid surface tension (GO:0050828)	56	10	.92	> 5	+	1.00E-05
complement activation (GO:0006956)	45	6	.74	> 5	+	2.54E-02
protein folding (GO:0006457)	155	20	2.54	> 5	+	6.56E-10
cellular component morphogenesis (GO:0032989)	478	43	7.83	> 5	+	7.33E-17
cell-matrix adhesion (GO:0007160)	89	8	1.46	> 5	+	2.93E-02
anatomical structure morphogenesis (GO:0009653)	596	50	9.76	> 5	+	1.29E-18
macrophage activation (GO:0042116)	167	13	2.74	4.75	+	1.19E-03
homeostatic process (GO:0042592)	209	16	3.42	4.67	+	1.23E-04
monosaccharide metabolic process (GO:0005996)	143	10	2.34	4.27	+	3.40E-02
receptor-mediated endocytosis (GO:0006898)	205	13	3.36	3.87	+	9.82E-03
cell-cell adhesion (GO:0016337)	391	24	6.41	3.75	+	1.14E-05
biological adhesion (GO:0022610)	606	36	9.93	3.63	+	1.09E-08
cell adhesion (GO:0007155)	579	32	9.49	3.37	+	7.70E-07
endocytosis (GO:0006897)	373	20	6.11	3.27	+	1.13E-03
generation of precursor metabolites and energy (GO:0006091)	274	14	4.49	3.12	+	4.78E-02
cellular component organization (GO:0016043)	1206	57	19.76	2.88	+	1.70E-10
cellular component organization or biogenesis (GO:0071840)	1316	62	21.56	2.88	+	1.60E-11
translation (GO:0006412)	435	20	7.13	2.81	+	9.82E-03
proteolysis (GO:0006508)	719	32	11.78	2.72	+	1.01E-04
protein metabolic process (GO:0019538)	2692	95	44.10	2.15	+	4.62E-11
developmental process (GO:0032502)	2456	86	40.24	2.14	+	1.46E-09
immune system process (GO:0002376)	1391	46	22.79	2.02	+	1.17E-03
cellular process (GO:0009987)	6708	169	109.90	1.54	+	5.48E-09
nucleobase-containing compound metabolic process (GO:0006139)	3467	31	56.80	.55	-	9.11E-03

Unclassified	8629	66	141.37	.47	-	0.00E00
RNA metabolic process (GO:0016070)	2360	14	38.66	.36	-	3.87E-04
transcription from RNA polymerase II promoter (GO:0006366)	1723	8	28.23	.28	-	8.70E-04
regulation of transcription from RNA polymerase II promoter (GO:0006357)	1319	6	21.61	.28	-	1.19E-02
transcription, DNA-dependent (GO:0006351)	1941	8	31.80	.25	-	4.74E-05
regulation of nucleobase-containing compound metabolic process (GO:0019219)	1700	7	27.85	.25	-	3.02E-04

© Copyright 2016 Paul Thomas All Rights Reserved.

GENEONTOLOGY Unifying Biology | PANTHER Classification System

LOGIN | REGISTER | CONTACT US

Home | About | PANTHER Data | PANTHER Tools | Workspace | Downloads | Help/Tutorial

Now includes comprehensive GO annotations directly imported from the GO database

Analysis Summary: Please report in publication **BM MSC REP MOLECULAR FUNCTION**

Analysis Type: PANTHER Overrepresentation Test (release 20150430)

Annotation Version and Release Date: PANTHER version 10.0 Released 2015-05-15

Analyzed List: Client Text Box Input (Homo sapiens) Change

Reference List: Homo sapiens (all genes in database) Change

Annotation Data Set: PANTHER GO-Slim Molecular Function

☑ Use the Bonferroni correction for multiple testing

Results

	Reference list	Client Text Box Input
Mapped IDs:	20814	341
Unmapped IDs:	0	75

Export results View: -- Please select a chart to display --

Displaying only results with P<0.05; click here to display all results

	Homo sapiens (REF)	Client Text Box Input				
PANTHER GO-Slim Molecular Function	#	#	expected	Fold Enrichment	+/-	P value
peroxidase activity (GO:0004601)	27	6	.44	> 5	+	1.16E-03
antioxidant activity (GO:0016209)	30	6	.49	> 5	+	2.10E-03
extracellular matrix structural constituent (GO:0005201)	76	12	1.25	> 5	+	1.33E-06
actin binding (GO:0003779)	176	19	2.88	> 5	+	3.43E-08
cytoskeletal protein binding (GO:0008092)	251	20	4.11	4.86	+	1.88E-06
peptidase inhibitor activity (GO:0030414)	226	18	3.70	4.86	+	1.03E-05
isomerase activity (GO:0016853)	166	13	2.72	4.78	+	8.51E-04
structural constituent of cytoskeleton (GO:0005200)	663	50	10.86	4.60	+	7.90E-17
serine-type peptidase activity (GO:0008236)	322	24	5.28	4.55	+	2.28E-07
structural molecule activity (GO:0005198)	1034	75	16.94	4.43	+	2.39E-25
metallopeptidase activity (GO:0008237)	170	11	2.79	3.95	+	2.41E-02
peptidase activity (GO:0008233)	630	39	10.32	3.78	+	3.61E-10
enzyme inhibitor activity (GO:0004857)	362	18	5.93	3.04	+	6.75E-03
oxidoreductase activity (GO:0016491)	626	31	10.26	3.02	+	1.21E-05
protein binding (GO:0005515)	2819	78	46.18	1.69	+	3.48E-04
catalytic activity (GO:0003824)	5209	117	85.34	1.37	+	1.37E-02
Unclassified	10020	93	164.16	.57	-	0.00E00
DNA binding (GO:0003677)	1878	8	30.77	.26	-	8.53E-05
sequence-specific DNA binding transcription factor activity (GO:0003700)	1462	4	23.95	< 0.2	-	5.87E-05
nucleic acid binding transcription factor activity (GO:0001071)	1646	4	26.97	< 0.2	-	3.70E-06

© Copyright 2016 Paul Thomas All Rights Reserved.

Now includes comprehensive GO annotations directly imported from the GO database

Analysis Summary: Please report in publication **BM MSC DOXO BIOLOGICAL PROCESS**

Analysis Type: PANTHER Overrepresentation Test (release 20150430)

Annotation Version and Release Date: PANTHER version 10.0 Released 2015-05-15

Analyzed List: Client Text Box Input (Homo sapiens) Change

Reference List: Homo sapiens (all genes in database) Change

Annotation Data Set: PANTHER GO-Slim Biological Process

☑ Use the Bonferroni correction for multiple testing

Results

	Reference list	Client Text Box Input
Mapped IDs:	20814	233
Unmapped IDs:	0	46

Export results View: -- Please select a chart to display --

Displaying only results with P<0.05; click here to display all results

	Homo sapiens (REF)	Client Text Box Input				
PANTHER GO-Slim Biological Process	#	#	expected	Fold Enrichment	+/-	P value
pentose-phosphate shunt (GO:0006098)	9	3	.10	> 5	+	3.45E-02
glycolysis (GO:0006096)	25	7	.28	> 5	+	4.25E-06
tricarboxylic acid cycle (GO:0006099)	20	4	.22	> 5	+	1.89E-02
protein folding (GO:0006457)	155	20	1.74	> 5	+	5.29E-13
monosaccharide metabolic process (GO:0005996)	143	12	1.60	> 5	+	2.42E-05
cellular component morphogenesis (GO:0032989)	478	26	5.35	4.86	+	1.11E-08
anatomical structure morphogenesis (GO:0009653)	596	31	6.67	4.65	+	4.11E-10
generation of precursor metabolites and energy (GO:0006091)	274	13	3.07	4.24	+	3.66E-03
cellular component organization or biogenesis (GO:0071840)	1316	44	14.73	2.99	+	1.66E-08
cellular component organization (GO:0016043)	1206	39	13.50	2.89	+	5.98E-07
carbohydrate metabolic process (GO:0005975)	573	18	6.41	2.81	+	2.14E-02
response to stress (GO:0006950)	659	20	7.38	2.71	+	1.40E-02
protein metabolic process (GO:0019538)	2692	55	30.14	1.83	+	1.38E-03
metabolic process (GO:0008152)	8247	125	92.32	1.35	+	2.29E-03
Unclassified	8629	43	96.60	.45	-	0.00E00
RNA metabolic process (GO:0016070)	2360	7	26.42	.26	-	7.21E-04
transcription from RNA polymerase II promoter (GO:0006366)	1723	4	19.29	.21	-	3.87E-03
regulation of transcription from RNA polymerase II promoter (GO:0006357)	1319	3	14.77	.20	-	4.12E-02
transcription, DNA-dependent (GO:0006351)	1941	4	21.73	< 0.2	-	4.38E-04
regulation of nucleobase-containing compound metabolic process (GO:0019219)	1700	3	19.03	< 0.2	-	8.97E-04

© Copyright 2016 Paul Thomas All Rights Reserved.

Now includes comprehensive GO annotations directly imported from the GO database

Analysis Summary: Please report in publication **BM MSC DOXO MOLECULAR FUNCTION**

Analysis Type: PANTHER Overrepresentation Test (release 20150430)

Annotation Version and Release Date: PANTHER version 10.0 Released 2015-05-15

Analyzed List: Client Text Box Input (Homo sapiens) Change

Reference List: Homo sapiens (all genes in database) Change

Annotation Data Set: PANTHER GO-Slim Molecular Function

Use the Bonferroni correction for multiple testing

Results

	Reference list	Client Text Box Input
Mapped IDs:	20814	233
Unmapped IDs:	0	46

Export results View: -- Please select a chart to display --

Displaying only results with P<0.05; click here to display all results

	Homo sapiens (REF)	Client Text Box Input				
PANTHER GO-Slim Molecular Function	#	#	expected	Fold Enrichment	+/-	P value
peroxidase activity (GO:0004601)	27	5	.30	> 5	+	2.66E-03
antioxidant activity (GO:0016209)	30	5	.34	> 5	+	4.39E-03
translation elongation factor activity (GO:0003746)	46	5	.51	> 5	+	3.22E-02
actin binding (GO:0003779)	176	15	1.97	> 5	+	3.79E-07
isomerase activity (GO:0016853)	166	14	1.86	> 5	+	1.49E-06
cytoskeletal protein binding (GO:0008092)	251	15	2.81	> 5	+	3.72E-05
peptidase inhibitor activity (GO:0030414)	226	13	2.53	> 5	+	3.66E-04
structural constituent of cytoskeleton (GO:0005200)	663	36	7.42	4.85	+	1.18E-12
oxidoreductase activity (GO:0016491)	626	29	7.01	4.14	+	2.57E-08
structural molecule activity (GO:0005198)	1034	41	11.57	3.54	+	3.56E-10
enzyme inhibitor activity (GO:0004857)	362	13	4.05	3.21	+	4.43E-02
peptidase activity (GO:0008233)	630	21	7.05	2.98	+	1.79E-03
protein binding (GO:0005515)	2819	52	31.56	1.65	+	3.01E-02
catalytic activity (GO:0003824)	5209	93	58.31	1.59	+	7.11E-05
Unclassified	10020	70	112.17	.62	-	0.00E00
sequence-specific DNA binding transcription factor activity (GO:0003700)	1462	4	16.37	.24	-	3.58E-02
nucleic acid binding transcription factor activity (GO:0001071)	1646	4	18.43	.22	-	6.27E-03

© Copyright 2016 Paul Thomas All Rights Reserved.

Now includes comprehensive GO annotations directly imported from the GO database

Analysis Summary: Please report in publication ? **BM MSC H2O2 BIOLOGICAL PROCESS**

Analysis Type: PANTHER Overrepresentation Test (release 20150430)

Annotation Version and Release Date: PANTHER version 10.0 Released 2015-05-15

Analyzed List: Client Text Box Input (Homo sapiens) Change

Reference List: Homo sapiens (all genes in database) Change

Annotation Data Set: PANTHER GO-Slim Biological Process

Use the Bonferroni correction for multiple testing ?

Results ?

	Reference list	Client Text Box Input
Mapped IDs:	20814	253
Unmapped IDs:	0	54

Export results View: -- Please select a chart to display --

Displaying only results with P<0.05; click here to display all results

	Homo sapiens (REF)	Client Text Box Input				
PANTHER GO-Slim Biological Process	#	#	expected	Fold Enrichment	+/-	P value
glycolysis (GO:0006096)	25	6	.30	> 5	+	1.77E-04
tricarboxylic acid cycle (GO:0006099)	20	4	.24	> 5	+	2.60E-02
protein folding (GO:0006457)	155	25	1.88	> 5	+	6.14E-18
regulation of liquid surface tension (GO:0050828)	56	7	.68	> 5	+	1.53E-03
protein complex assembly (GO:0006461)	107	13	1.30	> 5	+	2.52E-07
protein complex biogenesis (GO:0070271)	108	13	1.31	> 5	+	2.81E-07
monosaccharide metabolic process (GO:0005996)	143	12	1.74	> 5	+	5.86E-05
cellular component morphogenesis (GO:0032989)	478	32	5.81	> 5	+	2.15E-12
homeostatic process (GO:0042592)	209	13	2.54	> 5	+	5.10E-04
anatomical structure morphogenesis (GO:0009653)	596	37	7.24	> 5	+	1.50E-13
cellular component biogenesis (GO:0044085)	310	16	3.77	4.25	+	3.87E-04
generation of precursor metabolites and energy (GO:0006091)	274	14	3.33	4.20	+	1.93E-03
cell-cell adhesion (GO:0016337)	391	19	4.75	4.00	+	1.00E-04
cellular component organization or biogenesis (GO:0071840)	1316	58	16.00	3.63	+	2.01E-15
cell adhesion (GO:0007155)	579	24	7.04	3.41	+	5.31E-05
biological adhesion (GO:0022610)	606	25	7.37	3.39	+	3.21E-05
cellular component organization (GO:0016043)	1206	46	14.66	3.14	+	1.32E-09
carbohydrate metabolic process (GO:0005975)	573	21	6.96	3.02	+	2.02E-03
protein metabolic process (GO:0019538)	2692	76	32.72	2.32	+	1.83E-10
developmental process (GO:0032502)	2456	55	29.85	1.84	+	1.22E-03
cellular process (GO:0009987)	6708	120	81.54	1.47	+	7.80E-05
primary metabolic process (GO:0044238)	6825	117	82.96	1.41	+	1.31E-03
metabolic process (GO:0008152)	8247	137	100.24	1.37	+	4.63E-04
Unclassified	8629	41	104.89	.39	-	0.00E00
RNA metabolic process (GO:0016070)	2360	10	28.69	.35	-	5.52E-03
transcription from RNA polymerase II promoter (GO:0006366)	1723	5	20.94	.24	-	4.46E-03

transcription, DNA-dependent (GO:0006351)	1941	5	23.59	.21	-	4.55E-04
regulation of nucleobase-containing compound metabolic process (GO:0019219)	1700	4	20.66	< 0.2	-	1.21E-03
regulation of transcription from RNA polymerase II promoter (GO:0006357)	1319	2	16.03	< 0.2	-	2.32E-03

About | Release Information | Contact Us | System Requirements | Privacy Policy | Disclaimer
© Copyright 2016 Paul Thomas All Rights Reserved.

GENEONTOLOGY Unifying Biology   PANTHER Classification System

LOGIN   REGISTER   CONTACT US

Home | About | PANTHER Data | PANTHER Tools | Workspace | Downloads | Help/Tutorial

Now includes comprehensive GO annotations directly imported from the GO database

Analysis Summary: Please report in publication   **BM MSC H2O2 MOLECULAR FUNCTION**

Analysis Type: PANTHER Overrepresentation Test (release 20150430)

Annotation Version and Release Date: PANTHER version 10.0 Released 2015-05-15

Analyzed List: Client Text Box Input (Homo sapiens)   Change

Reference List: Homo sapiens (all genes in database)   Change

Annotation Data Set: PANTHER GO-Slim Molecular Function

Use the Bonferroni correction for multiple testing

Results

	Reference list	Client Text Box Input
Mapped IDs:	20814	253
Unmapped IDs:	0	54

Export results   View: -- Please select a chart to display --

Displaying only results with P<0.05; click here to display all results

	Homo sapiens (REF)	Client Text Box Input				
PANTHER GO-Slim Molecular Function	#	#	expected	Fold Enrichment	+/-	P value
peroxidase activity (GO:0004601)	27	6	.33	> 5	+	2.10E-04
antioxidant activity (GO:0016209)	30	6	.36	> 5	+	3.83E-04
translation elongation factor activity (GO:0003746)	46	6	.56	> 5	+	4.24E-03
extracellular matrix structural constituent (GO:0005201)	76	8	.92	> 5	+	9.00E-04
isomerase activity (GO:0016853)	166	15	2.02	> 5	+	5.34E-07
actin binding (GO:0003779)	176	15	2.14	> 5	+	1.15E-06
cytoskeletal protein binding (GO:0008092)	251	15	3.05	4.92	+	1.06E-04
structural constituent of cytoskeleton (GO:0005200)	663	39	8.06	4.84	+	9.91E-14
oxidoreductase activity (GO:0016491)	626	31	7.61	4.07	+	9.16E-09
structural molecule activity (GO:0005198)	1034	51	12.57	4.06	+	2.39E-15
peptidase inhibitor activity (GO:0030414)	226	11	2.75	4.00	+	2.08E-02
serine-type peptidase activity (GO:0008236)	322	14	3.91	3.58	+	8.49E-03
peptidase activity (GO:0008233)	630	24	7.66	3.13	+	1.78E-04
catalytic activity (GO:0003824)	5209	99	63.32	1.56	+	9.23E-05
Unclassified	10020	74	121.80	.61	-	0.00E00
DNA binding (GO:0003677)	1878	5	22.83	.22	-	6.80E-04
sequence-specific DNA binding transcription factor activity (GO:0003700)	1462	2	17.77	< 0.2	-	3.40E-04
nucleic acid binding transcription factor activity (GO:0001071)	1646	2	20.01	< 0.2	-	3.86E-05

About | Release Information | Contact Us | System Requirements | Privacy Policy | Disclaimer

© Copyright 2016 Paul Thomas All Rights Reserved.

Now includes comprehensive GO annotations directly imported from the GO database

Analysis Summary: Please report in publication **BM MSC IRL BIOLOGICAL PROCESS**

Analysis Type: PANTHER Overrepresentation Test (release 20150430)

Annotation Version and Release Date: PANTHER version 10.0 Released 2015-05-15

Analyzed List: Client Text Box Input (Homo sapiens) Change

Reference List: Homo sapiens (all genes in database) Change

Annotation Data Set: PANTHER GO-Slim Biological Process

Use the Bonferroni correction for multiple testing

Results

	Reference list	Client Text Box Input
Mapped IDs:	20814	524
Unmapped IDs:	0	116

Export results View: -- Please select a chart to display --

Displaying only results with P<0.05; click here to display all results

	Homo sapiens (REF)	Client Text Box Input				
PANTHER GO-Slim Biological Process	#	#	expected	Fold Enrichment	+/-	P value
tricarboxylic acid cycle (GO:0006099)	20	6	.50	> 5	+	3.18E-03
glycolysis (GO:0006096)	25	7	.63	> 5	+	9.59E-04
protein folding (GO:0006457)	155	33	3.90	> 5	+	8.46E-18
regulation of liquid surface tension (GO:0050828)	56	9	1.41	> 5	+	3.62E-03
protein complex assembly (GO:0006461)	107	17	2.69	> 5	+	8.48E-07
protein complex biogenesis (GO:0070271)	108	17	2.72	> 5	+	9.71E-07
cell-matrix adhesion (GO:0007160)	89	14	2.24	> 5	+	2.25E-05
complement activation (GO:0006956)	45	7	1.13	> 5	+	3.82E-02
regulation of translation (GO:0006417)	148	15	3.73	4.03	+	1.74E-03
translation (GO:0006412)	435	44	10.95	4.02	+	3.61E-12
cellular component morphogenesis (GO:0032989)	478	45	12.03	3.74	+	2.13E-11
monosaccharide metabolic process (GO:0005996)	143	13	3.60	3.61	+	2.04E-02
macrophage activation (GO:0042116)	167	15	4.20	3.57	+	6.93E-03
anatomical structure morphogenesis (GO:0009653)	596	51	15.00	3.40	+	1.64E-11
generation of precursor metabolites and energy (GO:0006091)	274	23	6.90	3.33	+	1.84E-04
homeostatic process (GO:0042592)	209	17	5.26	3.23	+	7.23E-03
cell-cell adhesion (GO:0016337)	391	30	9.84	3.05	+	2.72E-05
cellular component biogenesis (GO:0044085)	310	23	7.80	2.95	+	1.38E-03
proteolysis (GO:0006508)	719	51	18.10	2.82	+	1.42E-08
biological adhesion (GO:0022610)	606	42	15.26	2.75	+	1.44E-06
cell adhesion (GO:0007155)	579	40	14.58	2.74	+	3.64E-06
endocytosis (GO:0006897)	373	25	9.39	2.66	+	3.03E-03
protein metabolic process (GO:0019538)	2692	175	67.77	2.58	+	3.74E-31
cellular component organization or biogenesis (GO:0071840)	1316	82	33.13	2.48	+	1.18E-11
carbohydrate metabolic process (GO:0005975)	573	35	14.43	2.43	+	4.71E-04
cellular component movement (GO:0006928)	476	28	11.98	2.34	+	9.42E-03

cellular component organization (GO:0016043)	1206	66	30.36	2.17	+	8.83E-07
vesicle-mediated transport (GO:0016192)	895	44	22.53	1.95	+	5.62E-03
intracellular protein transport (GO:0006886)	1052	50	26.48	1.89	+	3.75E-03
protein transport (GO:0015031)	1082	51	27.24	1.87	+	3.82E-03
developmental process (GO:0032502)	2456	100	61.83	1.62	+	2.16E-04
primary metabolic process (GO:0044238)	6825	255	171.82	1.48	+	8.88E-12
metabolic process (GO:0008152)	8247	297	207.62	1.43	+	5.47E-13
cellular process (GO:0009987)	6708	231	168.88	1.37	+	2.11E-06
RNA metabolic process (GO:0016070)	2360	25	59.41	.42	-	2.38E-05
Unclassified	8629	89	217.24	.41	-	0.00E00
transcription from RNA polymerase II promoter (GO:0006366)	1723	12	43.38	.28	-	1.47E-06
transcription, DNA-dependent (GO:0006351)	1941	12	48.87	.25	-	1.65E-08
regulation of nucleobase-containing compound metabolic process (GO:0019219)	1700	8	42.80	< 0.2	-	5.67E-09
regulation of transcription from RNA polymerase II promoter (GO:0006357)	1319	6	33.21	< 0.2	-	8.96E-07

© Copyright 2016 Paul Thomas All Rights Reserved.

GENEONTOLOGY Unifying Biology

PANTHER Classification System

LOGIN REGISTER CONTACT US

Home | About | PANTHER Data | PANTHER Tools | Workspace | Downloads | Help/Tutorial

Now includes comprehensive GO annotations directly imported from the GO database

Analysis Summary: Please report in publication ? **BM MSC IRL MOLECULAR FUNCTION**

Analysis Type: PANTHER Overrepresentation Test (release 20150430)

Annotation Version and Release Date: PANTHER version 10.0 Released 2015-05-15

Analyzed List: Client Text Box Input (Homo sapiens) Change

Reference List: Homo sapiens (all genes in database) Change

Annotation Data Set: PANTHER GO-Slim Molecular Function

☑ Use the Bonferroni correction for multiple testing ?

Results ?

	Reference list	Client Text Box Input
Mapped IDs:	20814	524
Unmapped IDs:	0	116

Export results View: -- Please select a chart to display --

Displaying only results with P<0.05; click here to display all results

	Homo sapiens (REF)	Client Text Box Input				
PANTHER GO-Slim Molecular Function	#	#	expected	Fold Enrichment	+/-	P value
peroxidase activity (GO:0004601)	27	6	.68	> 5	+	1.27E-02
antioxidant activity (GO:0016209)	30	6	.76	> 5	+	2.24E-02
translation elongation factor activity (GO:0003746)	46	8	1.16	> 5	+	4.69E-03
actin binding (GO:0003779)	176	24	4.43	> 5	+	9.02E-09
extracellular matrix structural constituent (GO:0005201)	76	10	1.91	> 5	+	5.15E-03
isomerase activity (GO:0016853)	166	20	4.18	4.79	+	2.77E-06
structural constituent of ribosome (GO:0003735)	180	21	4.53	4.63	+	2.10E-06
serine-type endopeptidase inhibitor activity (GO:0004867)	81	9	2.04	4.41	+	4.43E-02
cytoskeletal protein binding (GO:0008092)	251	26	6.32	4.11	+	4.47E-07
translation factor activity, nucleic acid binding (GO:0008135)	136	14	3.42	4.09	+	2.24E-03
translation regulator activity (GO:0045182)	128	13	3.22	4.03	+	5.18E-03
peptidase inhibitor activity (GO:0030414)	226	21	5.69	3.69	+	8.68E-05
structural molecule activity (GO:0005198)	1034	96	26.03	3.69	+	5.35E-26
structural constituent of cytoskeleton (GO:0005200)	663	58	16.69	3.47	+	8.25E-14
oxidoreductase activity (GO:0016491)	626	52	15.76	3.30	+	2.17E-11
serine-type peptidase activity (GO:0008236)	322	23	8.11	2.84	+	1.91E-03
peptidase activity (GO:0008233)	630	45	15.86	2.84	+	1.24E-07
RNA binding (GO:0003723)	454	28	11.43	2.45	+	3.18E-03
enzyme inhibitor activity (GO:0004857)	362	22	9.11	2.41	+	2.99E-02
protein binding (GO:0005515)	2819	116	70.97	1.63	+	9.67E-06
hydrolase activity (GO:0016787)	2205	86	55.51	1.55	+	5.41E-03
catalytic activity (GO:0003824)	5209	198	131.14	1.51	+	1.28E-08
Unclassified	10020	156	252.26	.62	-	0.00E00

DNA binding (GO:0003677)	1878	16	47.28	.34	-	7.93E-06
sequence-specific DNA binding transcription factor activity (GO:0003700)	1462	8	36.81	.22	-	7.97E-07
nucleic acid binding transcription factor activity (GO:0001071)	1646	8	41.44	< 0.2	-	1.45E-08
protein kinase activity (GO:0004672)	443	1	11.15	< 0.2	-	2.66E-02

© Copyright 2016 Paul Thomas All Rights Reserved.

Now includes comprehensive GO annotations directly imported from the GO database

Analysis Summary: Please report in publication

BM MSC IRH BIOLOGICAL PROCESS

Analysis Type: PANTHER Overrepresentation Test (release 20150430)

Annotation Version and Release Date: PANTHER version 10.0 Released 2015-05-15

Analyzed List: Client Text Box Input (Homo sapiens) Change

Reference List: Homo sapiens (all genes in database) Change

Annotation Data Set: PANTHER GO-Slim Biological Process

☑ Use the Bonferroni correction for multiple testing

Results

	Reference list	Client Text Box Input
Mapped IDs:	20814	475
Unmapped IDs:	0	106

Export results View: -- Please select a chart to display --

Displaying only results with P<0.05; click here to display all results

	Homo sapiens (REF)	Client Text Box Input				
PANTHER GO-Slim Biological Process	#	#	expected	Fold Enrichment	+/-	P value
tricarboxylic acid cycle (GO:0006099)	20	6	.46	> 5	+	1.83E-03
glycolysis (GO:0006096)	25	7	.57	> 5	+	5.06E-04
complement activation (GO:0006956)	45	9	1.03	> 5	+	2.91E-04
protein folding (GO:0006457)	155	31	3.54	> 5	+	4.07E-17
regulation of liquid surface tension (GO:0050828)	56	9	1.28	> 5	+	1.67E-03
protein complex assembly (GO:0006461)	107	15	2.44	> 5	+	9.69E-06
protein complex biogenesis (GO:0070271)	108	15	2.46	> 5	+	1.09E-05
cell-matrix adhesion (GO:0007160)	89	10	2.03	4.92	+	1.10E-02
cellular component morphogenesis (GO:0032989)	478	44	10.91	4.03	+	2.82E-12
monosaccharide metabolic process (GO:0005996)	143	13	3.26	3.98	+	7.63E-03
generation of precursor metabolites and energy (GO:0006091)	274	23	6.25	3.68	+	3.38E-05
anatomical structure morphogenesis (GO:0009653)	596	50	13.60	3.68	+	1.38E-12
translation (GO:0006412)	435	36	9.93	3.63	+	1.47E-08
macrophage activation (GO:0042116)	167	13	3.81	3.41	+	3.52E-02
homeostatic process (GO:0042592)	209	16	4.77	3.35	+	7.81E-03
cell-cell adhesion (GO:0016337)	391	29	8.92	3.25	+	1.13E-05
cellular component biogenesis (GO:0044085)	310	22	7.07	3.11	+	9.35E-04
endocytosis (GO:0006897)	373	26	8.51	3.05	+	1.76E-04
cellular component organization or biogenesis (GO:0071840)	1316	81	30.03	2.70	+	1.30E-13
biological adhesion (GO:0022610)	606	37	13.83	2.68	+	2.24E-05
cell adhesion (GO:0007155)	579	35	13.21	2.65	+	6.32E-05
proteolysis (GO:0006508)	719	42	16.41	2.56	+	9.96E-06
protein metabolic process (GO:0019538)	2692	151	61.43	2.46	+	2.96E-24
cellular component organization (GO:0016043)	1206	66	27.52	2.40	+	1.48E-08
carbohydrate metabolic process (GO:0005975)	573	31	13.08	2.37	+	2.72E-03
vesicle-mediated transport (GO:0016192)	895	44	20.42	2.15	+	4.86E-04

protein transport (GO:0015031)	1082	50	24.69	2.02	+	5.42E-04
intracellular protein transport (GO:0006886)	1052	47	24.01	1.96	+	2.62E-03
immune system process (GO:0002376)	1391	54	31.74	1.70	+	2.50E-02
developmental process (GO:0032502)	2456	95	56.05	1.69	+	4.57E-05
transport (GO:0006810)	2473	88	56.44	1.56	+	3.89E-03
localization (GO:0051179)	2607	90	59.49	1.51	+	9.47E-03
cellular process (GO:0009987)	6708	227	153.08	1.48	+	3.01E-10
primary metabolic process (GO:0044238)	6825	226	155.75	1.45	+	3.74E-09
metabolic process (GO:0008152)	8247	271	188.21	1.44	+	2.98E-12
RNA metabolic process (GO:0016070)	2360	25	53.86	.46	-	7.48E-04
Unclassified	8629	75	196.92	.38	-	0.00E00
transcription from RNA polymerase II promoter (GO:0006366)	1723	11	39.32	.28	-	8.88E-06
transcription, DNA-dependent (GO:0006351)	1941	11	44.30	.25	-	1.53E-07
regulation of nucleobase-containing compound metabolic process (GO:0019219)	1700	6	38.80	< 0.2	-	5.21E-09
regulation of transcription from RNA polymerase II promoter (GO:0006357)	1319	4	30.10	< 0.2	-	3.45E-07

© Copyright 2016 Paul Thomas All Rights Reserved.

Now includes comprehensive GO annotations directly imported from the GO database

Analysis Summary: Please report in publication

BM MSC IRH MOLECULAR FUNCTION

Analysis Type: PANTHER Overrepresentation Test (release 20150430)

Annotation Version and Release Date: PANTHER version 10.0 Released 2015-05-15

Analyzed List: Client Text Box Input (Homo sapiens) Change

Reference List: Homo sapiens (all genes in database) Change

Annotation Data Set: PANTHER GO-Slim Molecular Function

☑ Use the Bonferroni correction for multiple testing

Results

	Reference list	Client Text Box Input
Mapped IDs:	20814	475
Unmapped IDs:	0	106

Export results View: -- Please select a chart to display --

Displaying only results with P<0.05; click here to display all results

	Homo sapiens (REF)	Client Text Box Input				
PANTHER GO-Slim Molecular Function	#	#	expected	Fold Enrichment	+/-	P value
peroxidase activity (GO:0004601)	27	7	.62	> 5	+	6.37E-04
antioxidant activity (GO:0016209)	30	7	.68	> 5	+	1.26E-03
extracellular matrix structural constituent (GO:0005201)	76	10	1.73	> 5	+	2.25E-03
actin binding (GO:0003779)	176	22	4.02	> 5	+	4.53E-08
isomerase activity (GO:0016853)	166	19	3.79	> 5	+	2.99E-06
cytoskeletal protein binding (GO:0008092)	251	24	5.73	4.19	+	1.26E-06
structural constituent of ribosome (GO:0003735)	180	17	4.11	4.14	+	2.30E-04
structural molecule activity (GO:0005198)	1034	93	23.60	3.94	+	1.98E-27
metallopeptidase activity (GO:0008237)	170	15	3.88	3.87	+	2.10E-03
structural constituent of cytoskeleton (GO:0005200)	663	58	15.13	3.83	+	9.19E-16
oxidoreductase activity (GO:0016491)	626	51	14.29	3.57	+	1.70E-12
peptidase inhibitor activity (GO:0030414)	226	18	5.16	3.49	+	1.17E-03
serine-type peptidase activity (GO:0008236)	322	24	7.35	3.27	+	1.14E-04
peptidase activity (GO:0008233)	630	42	14.38	2.92	+	1.88E-07
RNA binding (GO:0003723)	454	24	10.36	2.32	+	2.79E-02
protein binding (GO:0005515)	2819	109	64.33	1.69	+	3.34E-06
hydrolase activity (GO:0016787)	2205	83	50.32	1.65	+	7.00E-04
catalytic activity (GO:0003824)	5209	185	118.88	1.56	+	2.83E-09
binding (GO:0005488)	5733	167	130.83	1.28	+	2.89E-02
Unclassified	10020	128	228.67	.56	-	0.00E00
DNA binding (GO:0003677)	1878	15	42.86	.35	-	5.89E-05
sequence-specific DNA binding transcription factor activity (GO:0003700)	1462	6	33.36	< 0.2	-	5.49E-07
nucleic acid binding transcription factor activity (GO:0001071)	1646	6	37.56	< 0.2	-	1.24E-08

© Copyright 2016 Paul Thomas All Rights Reserved.

Now includes comprehensive GO annotations directly imported from the GO database

Analysis Summary: Please report in publication **A MSC REP BIOLOGICAL PROCESS**

Analysis Type: PANTHER Overrepresentation Test (release 20150430)

Annotation Version and Release Date: PANTHER version 10.0 Released 2015-05-15

Analyzed List: Client Text Box Input (Homo sapiens) Change

Reference List: Homo sapiens (all genes in database) Change

Annotation Data Set: PANTHER GO-Slim Biological Process

Use the Bonferroni correction for multiple testing

Results

	Reference list	Client Text Box Input
Mapped IDs:	20814	274
Unmapped IDs:	0	55

Export results View: -- Please select a chart to display --

Displaying only results with P<0.05; click here to display all results

	Homo sapiens (REF)	Client Text Box Input				
PANTHER GO-Slim Biological Process	#	#	expected	Fold Enrichment	+/-	P value
tricarboxylic acid cycle (GO:0006099)	20	5	.26	> 5	+	1.81E-03
glycolysis (GO:0006096)	25	6	.33	> 5	+	2.80E-04
regulation of liquid surface tension (GO:0050828)	56	7	.74	> 5	+	2.57E-03
protein folding (GO:0006457)	155	18	2.04	> 5	+	1.20E-09
cell-matrix adhesion (GO:0007160)	89	8	1.17	> 5	+	6.42E-03
monosaccharide metabolic process (GO:0005996)	143	9	1.88	4.78	+	3.11E-02
homeostatic process (GO:0042592)	209	13	2.75	4.73	+	1.21E-03
regulation of translation (GO:0006417)	148	9	1.95	4.62	+	4.00E-02
cellular component morphogenesis (GO:0032989)	478	29	6.29	4.61	+	3.28E-09
anatomical structure morphogenesis (GO:0009653)	596	35	7.85	4.46	+	5.44E-11
cell-cell adhesion (GO:0016337)	391	22	5.15	4.27	+	3.96E-06
translation (GO:0006412)	435	21	5.73	3.67	+	1.02E-04
generation of precursor metabolites and energy (GO:0006091)	274	13	3.61	3.60	+	1.93E-02
biological adhesion (GO:0022610)	606	28	7.98	3.51	+	2.89E-06
cell adhesion (GO:0007155)	579	26	7.62	3.41	+	1.71E-05
proteolysis (GO:0006508)	719	26	9.47	2.75	+	9.31E-04
cellular component organization or biogenesis (GO:0071840)	1316	47	17.32	2.71	+	1.13E-07
cellular component organization (GO:0016043)	1206	42	15.88	2.65	+	2.22E-06
protein metabolic process (GO:0019538)	2692	78	35.44	2.20	+	1.87E-09
immune system process (GO:0002376)	1391	37	18.31	2.02	+	8.93E-03
developmental process (GO:0032502)	2456	62	32.33	1.92	+	7.70E-05
cellular process (GO:0009987)	6708	136	88.31	1.54	+	3.88E-07
primary metabolic process (GO:0044238)	6825	119	89.85	1.32	+	3.35E-02
metabolic process (GO:0008152)	8247	140	108.57	1.29	+	1.74E-02
Unclassified	8629	48	113.59	.42	-	0.00E00
RNA metabolic process (GO:0016070)	2360	9	31.07	.29	-	2.72E-04

regulation of nucleobase-containing compound metabolic process (GO:0019219)	1700	3	22.38	< 0.2	-	4.34E-05
regulation of transcription from RNA polymerase II promoter (GO:0006357)	1319	2	17.36	< 0.2	-	6.83E-04
transcription from RNA polymerase II promoter (GO:0006366)	1723	2	22.68	< 0.2	-	3.81E-06
transcription, DNA-dependent (GO:0006351)	1941	2	25.55	< 0.2	-	2.11E-07

© Copyright 2016 Paul Thomas All Rights Reserved.

Now includes comprehensive GO annotations directly imported from the GO database

Analysis Summary: Please report in publication ? **A MSC REP MOLECULAR FUNCTION**

Analysis Type: PANTHER Overrepresentation Test (release 20150430)

Annotation Version and Release Date: PANTHER version 10.0 Released 2015-05-15

Analyzed List: Client Text Box Input (Homo sapiens) Change

Reference List: Homo sapiens (all genes in database) Change

Annotation Data Set: PANTHER GO-Slim Molecular Function

☑ Use the Bonferroni correction for multiple testing ?

Results ?

	Reference list	Client Text Box Input
Mapped IDs:	20814	274
Unmapped IDs:	0	55

Export results View: -- Please select a chart to display --

Displaying only results with P<0.05; click here to display all results

	Homo sapiens (REF)	Client Text Box Input				
PANTHER GO-Slim Molecular Function	#	#	expected	Fold Enrichment	+/-	P value
peroxidase activity (GO:0004601)	27	5	.36	> 5	+	5.76E-03
antioxidant activity (GO:0016209)	30	5	.39	> 5	+	9.45E-03
translation elongation factor activity (GO:0003746)	46	6	.61	> 5	+	6.61E-03
extracellular matrix structural constituent (GO:0005201)	76	9	1.00	> 5	+	1.72E-04
isomerase activity (GO:0016853)	166	17	2.19	> 5	+	2.44E-08
peptidase inhibitor activity (GO:0030414)	226	18	2.98	> 5	+	3.56E-07
actin binding (GO:0003779)	176	13	2.32	> 5	+	1.46E-04
translation regulator activity (GO:0045182)	128	9	1.69	> 5	+	1.04E-02
translation factor activity, nucleic acid binding (GO:0008135)	136	9	1.79	> 5	+	1.64E-02
metallopeptidase activity (GO:0008237)	170	10	2.24	4.47	+	1.77E-02
structural molecule activity (GO:0005198)	1034	58	13.61	4.26	+	1.29E-18
structural constituent of cytoskeleton (GO:0005200)	663	37	8.73	4.24	+	3.66E-11
cytoskeletal protein binding (GO:0008092)	251	14	3.30	4.24	+	1.38E-03
serine-type peptidase activity (GO:0008236)	322	17	4.24	4.01	+	3.08E-04
enzyme inhibitor activity (GO:0004857)	362	19	4.77	3.99	+	8.23E-05
peptidase activity (GO:0008233)	630	33	8.29	3.98	+	4.23E-09
oxidoreductase activity (GO:0016491)	626	32	8.24	3.88	+	1.59E-08
hydrolase activity (GO:0016787)	2205	52	29.03	1.79	+	4.29E-03
protein binding (GO:0005515)	2819	65	37.11	1.75	+	6.58E-04
catalytic activity (GO:0003824)	5209	103	68.57	1.50	+	4.84E-04
Unclassified	10020	77	131.91	.58	-	0.00E00
DNA binding (GO:0003677)	1878	7	24.72	.28	-	2.54E-03
sequence-specific DNA binding transcription factor activity (GO:0003700)	1462	2	19.25	< 0.2	-	8.56E-05

nucleic acid binding transcription factor activity (GO:0001071)	1646	2	21.67	< 0.2	-	7.98E-06

© Copyright 2016 Paul Thomas All Rights Reserved.

Now includes comprehensive GO annotations directly imported from the GO database

Analysis Summary: Please report in publication

A MSC DOXO BIOLOGICAL PROCESS

Analysis Type: PANTHER Overrepresentation Test (release 20150430)

Annotation Version and Release Date: PANTHER version 10.0 Released 2015-05-15

Analyzed List: Client Text Box Input (Homo sapiens) Change

Reference List: Homo sapiens (all genes in database) Change

Annotation Data Set: PANTHER GO-Slim Biological Process

Use the Bonferroni correction for multiple testing

Results

	Reference list	Client Text Box Input
Mapped IDs:	20814	728
Unmapped IDs:	0	148

Export results View: -- Please select a chart to display --

Displaying only results with P<0.05; click here to display all results

	Homo sapiens (REF)	Client Text Box Input				
PANTHER GO-Slim Biological Process	#	#	expected	Fold Enrichment	+/-	P value
tricarboxylic acid cycle (GO:0006099)	20	7	.70	> 5	+	1.91E-03
glycolysis (GO:0006096)	25	8	.87	> 5	+	8.38E-04
protein folding (GO:0006457)	155	39	5.42	> 5	+	1.09E-18
regulation of translation (GO:0006417)	148	26	5.18	> 5	+	1.05E-08
protein complex assembly (GO:0006461)	107	18	3.74	4.81	+	1.84E-05
protein complex biogenesis (GO:0070271)	108	18	3.78	4.77	+	2.11E-05
translation (GO:0006412)	435	70	15.21	4.60	+	3.94E-23
purine nucleobase metabolic process (GO:0006144)	88	14	3.08	4.55	+	9.28E-04
monosaccharide metabolic process (GO:0005996)	143	17	5.00	3.40	+	4.01E-03
generation of precursor metabolites and energy (GO:0006091)	274	29	9.58	3.03	+	5.66E-05
cellular component biogenesis (GO:0044085)	310	30	10.84	2.77	+	2.19E-04
cellular component morphogenesis (GO:0032989)	478	45	16.72	2.69	+	1.00E-06
endocytosis (GO:0006897)	373	35	13.05	2.68	+	5.67E-05
receptor-mediated endocytosis (GO:0006898)	205	19	7.17	2.65	+	3.52E-02
anatomical structure morphogenesis (GO:0009653)	596	52	20.85	2.49	+	7.74E-07
vesicle-mediated transport (GO:0016192)	895	73	31.30	2.33	+	9.23E-09
protein metabolic process (GO:0019538)	2692	216	94.16	2.29	+	4.16E-30
cellular component organization or biogenesis (GO:0071840)	1316	105	46.03	2.28	+	1.12E-12
protein transport (GO:0015031)	1082	81	37.84	2.14	+	4.30E-08
cellular component movement (GO:0006928)	476	35	16.65	2.10	+	1.02E-02
carbohydrate metabolic process (GO:0005975)	573	42	20.04	2.10	+	1.96E-03
cellular component organization (GO:0016043)	1206	88	42.18	2.09	+	2.32E-08
intracellular protein transport (GO:0006886)	1052	75	36.80	2.04	+	1.67E-06
proteolysis (GO:0006508)	719	49	25.15	1.95	+	2.41E-03
transport (GO:0006810)	2473	130	86.50	1.50	+	3.73E-04
primary metabolic process (GO:0044238)	6825	358	238.71	1.50	+	1.02E-17

metabolic process (GO:0008152)	8247	432	288.45	1.50	+	1.35E-24
localization (GO:0051179)	2607	133	91.18	1.46	+	1.27E-03
cellular process (GO:0009987)	6708	287	234.62	1.22	+	5.67E-03
RNA metabolic process (GO:0016070)	2360	49	82.54	.59	-	4.15E-03
Unclassified	8629	120	301.81	.40	-	0.00E00
transcription from RNA polymerase II promoter (GO:0006366)	1723	20	60.26	.33	-	1.08E-07
transcription, DNA-dependent (GO:0006351)	1941	20	67.89	.29	-	3.28E-10
regulation of nucleobase-containing compound metabolic process (GO:0019219)	1700	15	59.46	.25	-	3.23E-10
regulation of transcription from RNA polymerase II promoter (GO:0006357)	1319	11	46.13	.24	-	5.46E-08

© Copyright 2016 Paul Thomas All Rights Reserved.

GENEONTOLOGY Unifying Biology
PANTHER Classification System
LOGIN REGISTER CONTACT US

Now includes comprehensive GO annotations directly imported from the GO database

Analysis Summary: Please report in publication

A MSC DOXO MOLECULAR FUNCTION

Analysis Type: PANTHER Overrepresentation Test (release 20150430)

Annotation Version and Release Date: PANTHER version 10.0 Released 2015-05-15

Analyzed List: Client Text Box Input (Homo sapiens) Change

Reference List: Homo sapiens (all genes in database) Change

Annotation Data Set: PANTHER GO-Slim Molecular Function

Use the Bonferroni correction for multiple testing

Results

	Reference list	Client Text Box Input
Mapped IDs:	20814	728
Unmapped IDs:	0	148

Export results View: -- Please select a chart to display --

Displaying only results with P<0.05; click here to display all results

	Homo sapiens (REF)	Client Text Box Input				
PANTHER GO-Slim Molecular Function	#	#	expected	Fold Enrichment	+/-	P value
aminoacyl-tRNA ligase activity (GO:0004812)	27	10	.94	> 5	+	1.06E-05
translation elongation factor activity (GO:0003746)	46	10	1.61	> 5	+	1.21E-03
translation factor activity, nucleic acid binding (GO:0008135)	136	27	4.76	> 5	+	2.23E-10
translation initiation factor activity (GO:0003743)	97	19	3.39	> 5	+	5.75E-07
translation regulator activity (GO:0045182)	128	24	4.48	> 5	+	1.23E-08
actin binding (GO:0003779)	176	30	6.16	4.87	+	5.46E-10
isomerase activity (GO:0016853)	166	28	5.81	4.82	+	3.65E-09
cytoskeletal protein binding (GO:0008092)	251	36	8.78	4.10	+	5.07E-10
structural constituent of ribosome (GO:0003735)	180	25	6.30	3.97	+	1.98E-06
cysteine-type peptidase activity (GO:0008234)	92	12	3.22	3.73	+	2.19E-02
RNA binding (GO:0003723)	454	51	15.88	3.21	+	1.46E-10
structural constituent of cytoskeleton (GO:0005200)	663	71	23.19	3.06	+	4.15E-14
structural molecule activity (GO:0005198)	1034	107	36.17	2.96	+	5.34E-21
oxidoreductase activity (GO:0016491)	626	62	21.90	2.83	+	1.02E-10
GTPase activity (GO:0003924)	262	24	9.16	2.62	+	4.72E-03
ligase activity (GO:0016874)	415	36	14.52	2.48	+	1.79E-04
catalytic activity (GO:0003824)	5209	298	182.19	1.64	+	7.00E-19
hydrolase activity (GO:0016787)	2205	119	77.12	1.54	+	2.48E-04
protein binding (GO:0005515)	2819	152	98.60	1.54	+	5.97E-06
binding (GO:0005488)	5733	255	200.52	1.27	+	1.02E-03
Unclassified	10020	232	350.46	.66	-	0.00E00
receptor activity (GO:0004872)	1636	28	57.22	.49	-	1.27E-03
DNA binding (GO:0003677)	1878	30	65.69	.46	-	4.35E-05

sequence-specific DNA binding transcription factor activity (GO:0003700)	1462	17	51.14	.33	-	2.06E-06
nucleic acid binding transcription factor activity (GO:0001071)	1646	17	57.57	.30	-	1.65E-08
protein kinase activity (GO:0004672)	443	3	15.49	< 0.2	-	2.14E-02

© Copyright 2016 Paul Thomas All Rights Reserved.

Now includes comprehensive GO annotations directly imported from the GO database

Analysis Summary: Please report in publication ?

A MSC H2O2 BIOLOGICAL PROCESS

Analysis Type: PANTHER Overrepresentation Test (release 20150430)

Annotation Version and Release Date: PANTHER version 10.0 Released 2015-05-15

Analyzed List: Client Text Box Input (Homo sapiens) Change

Reference List: Homo sapiens (all genes in database) Change

Annotation Data Set: PANTHER GO-Slim Biological Process

☑ Use the Bonferroni correction for multiple testing ?

Results ?

	Reference list	Client Text Box Input
Mapped IDs:	20814	297
Unmapped IDs:	0	52

Export results View: -- Please select a chart to display --

Displaying only results with P<0.05; click here to display all results

PANTHER GO-Slim Biological Process	Homo sapiens (REF) #	Client Text Box Input #	expected	Fold Enrichment	+/-	P value
tricarboxylic acid cycle (GO:0006099)	20	5	.29	> 5	+	2.67E-03
glycolysis (GO:0006096)	25	6	.36	> 5	+	4.46E-04
protein folding (GO:0006457)	155	27	2.21	> 5	+	1.75E-18
regulation of liquid surface tension (GO:0050828)	56	7	.80	> 5	+	4.31E-03
protein complex assembly (GO:0006461)	107	12	1.53	> 5	+	1.53E-05
protein complex biogenesis (GO:0070271)	108	12	1.54	> 5	+	1.69E-05
regulation of translation (GO:0006417)	148	11	2.11	> 5	+	2.67E-03
macrophage activation (GO:0042116)	167	11	2.38	4.62	+	7.96E-03
cellular component morphogenesis (GO:0032989)	478	30	6.82	4.40	+	4.79E-09
anatomical structure morphogenesis (GO:0009653)	596	35	8.50	4.12	+	5.76E-10
generation of precursor metabolites and energy (GO:0006091)	274	14	3.91	3.58	+	1.13E-02
cell-cell adhesion (GO:0016337)	391	19	5.58	3.41	+	1.07E-03
proteolysis (GO:0006508)	719	32	10.26	3.12	+	4.24E-06
translation (GO:0006412)	435	19	6.21	3.06	+	4.66E-03
cell adhesion (GO:0007155)	579	23	8.26	2.78	+	2.86E-03
biological adhesion (GO:0022610)	606	24	8.65	2.78	+	1.94E-03
cellular component organization or biogenesis (GO:0071840)	1316	51	18.78	2.72	+	2.08E-08
carbohydrate metabolic process (GO:0005975)	573	22	8.18	2.69	+	7.32E-03
protein metabolic process (GO:0019538)	2692	96	38.41	2.50	+	1.00E-15
cellular component organization (GO:0016043)	1206	40	17.21	2.32	+	1.63E-04
immune system process (GO:0002376)	1391	45	19.85	2.27	+	6.03E-05
regulation of catalytic activity (GO:0050790)	1073	32	15.31	2.09	+	1.75E-02
regulation of molecular function (GO:0065009)	1096	32	15.64	2.05	+	2.57E-02
developmental process (GO:0032502)	2456	68	35.05	1.94	+	1.35E-05
primary metabolic process (GO:0044238)	6825	143	97.39	1.47	+	6.57E-06
metabolic process (GO:0008152)	8247	172	117.68	1.46	+	3.38E-08

cellular process (GO:0009987)	6708	137	95.72	1.43	+	9.25E-05
RNA metabolic process (GO:0016070)	2360	14	33.68	.42	-	1.15E-02
transcription from RNA polymerase II promoter (GO:0006366)	1723	9	24.59	.37	-	4.09E-02
Unclassified	8629	45	123.13	.37	-	0.00E00
transcription, DNA-dependent (GO:0006351)	1941	9	27.70	.32	-	4.15E-03
regulation of transcription from RNA polymerase II promoter (GO:0006357)	1319	5	18.82	.27	-	2.75E-02
regulation of nucleobase-containing compound metabolic process (GO:0019219)	1700	6	24.26	.25	-	1.30E-03
nitrogen compound metabolic process (GO:0006807)	1099	3	15.68	< 0.2	-	2.01E-02

© Copyright 2016 Paul Thomas All Rights Reserved.

GENEONTOLOGY Unifying Biology | PANTHER Classification System

LOGIN REGISTER CONTACT US

Home | About | PANTHER Data | PANTHER Tools | Workspace | Downloads | Help/Tutorial

Now includes comprehensive GO annotations directly imported from the GO database

Analysis Summary: Please report in publication ? **A MSC H2O2 MOLECULAR FUNCTION**

Analysis Type: PANTHER Overrepresentation Test (release 20150430)

Annotation Version and Release Date: PANTHER version 10.0 Released 2015-05-15

Analyzed List: Client Text Box Input (Homo sapiens) Change

Reference List: Homo sapiens (all genes in database) Change

Annotation Data Set: PANTHER GO-Slim Molecular Function

☑ Use the Bonferroni correction for multiple testing ?

Results ?

	Reference list	Client Text Box Input
Mapped IDs:	20814	297
Unmapped IDs:	0	52

Export results View: -- Please select a chart to display --

Displaying only results with P<0.05; click here to display all results

	Homo sapiens (REF)	Client Text Box Input				
PANTHER GO-Slim Molecular Function	#	#	expected	Fold Enrichment	+/-	P value
antioxidant activity (GO:0016209)	30	7	.43	> 5	+	5.71E-05
peroxidase activity (GO:0004601)	27	6	.39	> 5	+	5.28E-04
translation elongation factor activity (GO:0003746)	46	7	.66	> 5	+	9.37E-04
extracellular matrix structural constituent (GO:0005201)	76	9	1.08	> 5	+	3.33E-04
isomerase activity (GO:0016853)	166	17	2.37	> 5	+	8.41E-08
peptidase inhibitor activity (GO:0030414)	226	20	3.22	> 5	+	3.03E-08
serine-type endopeptidase inhibitor activity (GO:0004867)	81	7	1.16	> 5	+	3.23E-02
actin binding (GO:0003779)	176	15	2.51	> 5	+	9.64E-06
translation regulator activity (GO:0045182)	128	10	1.83	> 5	+	3.34E-03
translation factor activity, nucleic acid binding (GO:0008135)	136	10	1.94	> 5	+	5.55E-03
cytoskeletal protein binding (GO:0008092)	251	17	3.58	4.75	+	3.22E-05
structural constituent of cytoskeleton (GO:0005200)	663	39	9.46	4.12	+	2.09E-11
enzyme inhibitor activity (GO:0004857)	362	21	5.17	4.07	+	1.53E-05
structural molecule activity (GO:0005198)	1034	58	14.75	3.93	+	7.55E-17
peptidase activity (GO:0008233)	630	35	8.99	3.89	+	2.01E-09
serine-type peptidase activity (GO:0008236)	322	17	4.59	3.70	+	9.04E-04
oxidoreductase activity (GO:0016491)	626	32	8.93	3.58	+	1.24E-07
calcium ion binding (GO:0005509)	449	20	6.41	3.12	+	1.63E-03
RNA binding (GO:0003723)	454	19	6.48	2.93	+	6.30E-03
enzyme regulator activity (GO:0030234)	1002	31	14.30	2.17	+	8.95E-03
protein binding (GO:0005515)	2819	81	40.22	2.01	+	5.88E-08
hydrolase activity (GO:0016787)	2205	54	31.46	1.72	+	1.02E-02
catalytic activity (GO:0003824)	5209	119	74.33	1.60	+	1.51E-06

binding (GO:0005488)	5733	110	81.81	1.34	+	3.92E-02
Unclassified	10020	82	142.98	.57	-	0.00E00
DNA binding (GO:0003677)	1878	7	26.80	.26	-	4.97E-04
sequence-specific DNA binding transcription factor activity (GO:0003700)	1462	5	20.86	.24	-	3.98E-03
nucleic acid binding transcription factor activity (GO:0001071)	1646	5	23.49	.21	-	4.29E-04

© Copyright 2016 Paul Thomas All Rights Reserved.

Now includes comprehensive GO annotations directly imported from the GO database

Analysis Summary: Please report in publication ?   **A MSC IRL BIOLOGICAL PROCESS**

Analysis Type: PANTHER Overrepresentation Test (release 20150430)

Annotation Version and Release Date: PANTHER version 10.0 Released 2015-05-15

Analyzed List: Client Text Box Input (Homo sapiens) Change

Reference List: Homo sapiens (all genes in database) Change

Annotation Data Set: PANTHER GO-Slim Biological Process

☑ Use the Bonferroni correction for multiple testing ?

Results ?

	Reference list	Client Text Box Input
Mapped IDs:	20814	465
Unmapped IDs:	0	141

Export results   View: -- Please select a chart to display --

Displaying only results with P<0.05; click here to display all results

	Homo sapiens (REF)	Client Text Box Input				
PANTHER GO-Slim Biological Process	#	#	expected	Fold Enrichment	+/-	P value
glycolysis (GO:0006096)	25	7	.56	> 5	+	4.40E-04
tricarboxylic acid cycle (GO:0006099)	20	5	.45	> 5	+	2.22E-02
protein folding (GO:0006457)	155	31	3.46	> 5	+	2.21E-17
cell-matrix adhesion (GO:0007160)	89	12	1.99	> 5	+	2.57E-04
protein complex assembly (GO:0006461)	107	14	2.39	> 5	+	4.78E-05
protein complex biogenesis (GO:0070271)	108	14	2.41	> 5	+	5.34E-05
cellular component morphogenesis (GO:0032989)	478	49	10.68	4.59	+	5.20E-16
anatomical structure morphogenesis (GO:0009653)	596	57	13.32	4.28	+	1.67E-17
cell-cell adhesion (GO:0016337)	391	29	8.74	3.32	+	7.18E-06
generation of precursor metabolites and energy (GO:0006091)	274	19	6.12	3.10	+	4.27E-03
cellular component organization or biogenesis (GO:0071840)	1316	90	29.40	3.06	+	6.63E-19
biological adhesion (GO:0022610)	606	40	13.54	2.95	+	4.48E-07
cell adhesion (GO:0007155)	579	38	12.94	2.94	+	1.34E-06
cellular component organization (GO:0016043)	1206	77	26.94	2.86	+	3.39E-14
endocytosis (GO:0006897)	373	22	8.33	2.64	+	1.09E-02
translation (GO:0006412)	435	25	9.72	2.57	+	5.15E-03
proteolysis (GO:0006508)	719	39	16.06	2.43	+	1.12E-04
carbohydrate metabolic process (GO:0005975)	573	31	12.80	2.42	+	1.79E-03
protein metabolic process (GO:0019538)	2692	132	60.14	2.19	+	2.24E-16
vesicle-mediated transport (GO:0016192)	895	39	19.99	1.95	+	1.59E-02
protein transport (GO:0015031)	1082	45	24.17	1.86	+	1.31E-02
intracellular protein transport (GO:0006886)	1052	43	23.50	1.83	+	2.79E-02
developmental process (GO:0032502)	2456	99	54.87	1.80	+	1.02E-06
cellular process (GO:0009987)	6708	218	149.86	1.45	+	7.82E-09
metabolic process (GO:0008152)	8247	244	184.24	1.32	+	3.15E-06
primary metabolic process (GO:0044238)	6825	198	152.48	1.30	+	1.43E-03

nucleobase-containing compound metabolic process (GO:0006139)	3467	42	77.46	.54	-	3.33E-04
Unclassified	8629	82	192.78	.43	-	0.00E00
RNA metabolic process (GO:0016070)	2360	14	52.72	.27	-	9.38E-09
transcription from RNA polymerase II promoter (GO:0006366)	1723	7	38.49	< 0.2	-	4.08E-08
transcription, DNA-dependent (GO:0006351)	1941	7	43.36	< 0.2	-	4.77E-10
regulation of transcription from RNA polymerase II promoter (GO:0006357)	1319	4	29.47	< 0.2	-	6.11E-07
regulation of nucleobase-containing compound metabolic process (GO:0019219)	1700	5	37.98	< 0.2	-	1.54E-09

© Copyright 2016 Paul Thomas All Rights Reserved.

Now includes comprehensive GO annotations directly imported from the GO database

Analysis Summary: Please report in publication **A MSC IRL MOLECULAR FUNCTION**

Analysis Type: PANTHER Overrepresentation Test (release 20150430)

Annotation Version and Release Date: PANTHER version 10.0 Released 2015-05-15

Analyzed List: Client Text Box Input (Homo sapiens) Change

Reference List: Homo sapiens (all genes in database) Change

Annotation Data Set: PANTHER GO-Slim Molecular Function

☑ Use the Bonferroni correction for multiple testing

Results

	Reference list	Client Text Box Input
Mapped IDs:	20814	465
Unmapped IDs:	0	141

Export results View: -- Please select a chart to display --

Displaying only results with P<0.05; click here to display all results

	Homo sapiens (REF)	Client Text Box Input				
PANTHER GO-Slim Molecular Function	#	#	expected	Fold Enrichment	+/-	P value
antioxidant activity (GO:0016209)	30	9	.67	> 5	+	6.52E-06
peroxidase activity (GO:0004601)	27	8	.60	> 5	+	4.09E-05
translation elongation factor activity (GO:0003746)	46	7	1.03	> 5	+	1.61E-02
actin binding (GO:0003779)	176	23	3.93	> 5	+	4.95E-09
isomerase activity (GO:0016853)	166	17	3.71	4.58	+	5.79E-05
metallopeptidase activity (GO:0008237)	170	17	3.80	4.48	+	8.00E-05
structural constituent of cytoskeleton (GO:0005200)	663	66	14.81	4.46	+	1.36E-21
cytoskeletal protein binding (GO:0008092)	251	24	5.61	4.28	+	8.39E-07
structural molecule activity (GO:0005198)	1034	91	23.10	3.94	+	8.19E-27
peptidase inhibitor activity (GO:0030414)	226	17	5.05	3.37	+	3.27E-03
oxidoreductase activity (GO:0016491)	626	47	13.99	3.36	+	1.62E-10
serine-type peptidase activity (GO:0008236)	322	23	7.19	3.20	+	2.74E-04
peptidase activity (GO:0008233)	630	45	14.07	3.20	+	2.58E-09
hydrolase activity (GO:0016787)	2205	74	49.26	1.50	+	4.72E-02
protein binding (GO:0005515)	2819	93	62.98	1.48	+	1.24E-02
catalytic activity (GO:0003824)	5209	169	116.37	1.45	+	6.86E-06
Unclassified	10020	138	223.85	.62	-	0.00E00
DNA binding (GO:0003677)	1878	13	41.96	.31	-	1.09E-05
kinase activity (GO:0016301)	778	4	17.38	.23	-	1.89E-02
sequence-specific DNA binding transcription factor activity (GO:0003700)	1462	6	32.66	< 0.2	-	1.00E-06
nucleic acid binding transcription factor activity (GO:0001071)	1646	6	36.77	< 0.2	-	2.49E-08

© Copyright 2016 Paul Thomas All Rights Reserved.

Now includes comprehensive GO annotations directly imported from the GO database

Analysis Summary: Please report in publication ? A MSC IRH BIOLOGICAL PROCESS

Analysis Type: PANTHER Overrepresentation Test (release 20150430)

Annotation Version and Release Date: PANTHER version 10.0 Released 2015-05-15

Analyzed List: Client Text Box Input (Homo sapiens) Change

Reference List: Homo sapiens (all genes in database) Change

Annotation Data Set: PANTHER GO-Slim Biological Process

☑ Use the Bonferroni correction for multiple testing ?

Results ?

	Reference list	Client Text Box Input
Mapped IDs:	20814	400
Unmapped IDs:	0	106

Export results View: -- Please select a chart to display --

Displaying only results with P<0.05; click here to display all results

	Homo sapiens (REF)	Client Text Box Input				
PANTHER GO-Slim Biological Process	#	#	expected	Fold Enrichment	+/-	P value
tricarboxylic acid cycle (GO:0006099)	20	5	.38	> 5	+	1.10E-02
glycolysis (GO:0006096)	25	6	.48	> 5	+	2.43E-03
protein folding (GO:0006457)	155	28	2.98	> 5	+	3.54E-16
regulation of liquid surface tension (GO:0050828)	56	8	1.08	> 5	+	3.62E-03
cell-matrix adhesion (GO:0007160)	89	11	1.71	> 5	+	3.87E-04
protein complex assembly (GO:0006461)	107	12	2.06	> 5	+	3.56E-04
protein complex biogenesis (GO:0070271)	108	12	2.08	> 5	+	3.92E-04
cellular component morphogenesis (GO:0032989)	478	50	9.19	> 5	+	1.49E-19
anatomical structure morphogenesis (GO:0009653)	596	58	11.45	> 5	+	1.87E-21
cell-cell adhesion (GO:0016337)	391	31	7.51	4.13	+	1.35E-08
biological adhesion (GO:0022610)	606	41	11.65	3.52	+	1.35E-09
homeostatic process (GO:0042592)	209	14	4.02	3.49	+	1.56E-02
cell adhesion (GO:0007155)	579	38	11.13	3.42	+	1.98E-08
cellular component organization or biogenesis (GO:0071840)	1316	85	25.29	3.36	+	1.42E-20
cellular component organization (GO:0016043)	1206	74	23.18	3.19	+	2.46E-16
generation of precursor metabolites and energy (GO:0006091)	274	16	5.27	3.04	+	2.38E-02
endocytosis (GO:0006897)	373	21	7.17	2.93	+	3.56E-03
proteolysis (GO:0006508)	719	38	13.82	2.75	+	6.60E-06
carbohydrate metabolic process (GO:0005975)	573	25	11.01	2.27	+	3.48E-02
developmental process (GO:0032502)	2456	102	47.20	2.16	+	8.44E-12
protein metabolic process (GO:0019538)	2692	108	51.73	2.09	+	1.09E-11
immune system process (GO:0002376)	1391	51	26.73	1.91	+	1.90E-03
cellular process (GO:0009987)	6708	194	128.91	1.50	+	2.35E-09
metabolic process (GO:0008152)	8247	197	158.49	1.24	+	1.32E-02
nucleobase-containing compound metabolic process (GO:0006139)	3467	32	66.63	.48	-	7.03E-05
Unclassified	8629	73	165.83	.44	-	0.00E00

nitrogen compound metabolic process (GO:0006807)	1099	6	21.12	.28	-	1.85E-02
RNA metabolic process (GO:0016070)	2360	9	45.35	< 0.2	-	2.01E-09
regulation of transcription from RNA polymerase II promoter (GO:0006357)	1319	4	25.35	< 0.2	-	2.41E-05
regulation of nucleobase-containing compound metabolic process (GO:0019219)	1700	5	32.67	< 0.2	-	1.87E-07
transcription from RNA polymerase II promoter (GO:0006366)	1723	4	33.11	< 0.2	-	1.69E-08
transcription, DNA-dependent (GO:0006351)	1941	4	37.30	< 0.2	-	2.83E-10

© Copyright 2016 Paul Thomas All Rights Reserved.

Now includes comprehensive GO annotations directly imported from the GO database

Analysis Summary: Please report in publication ? **A MSC IRH MOLECULAR FUNCTION**

Analysis Type: PANTHER Overrepresentation Test (release 20150430)

Annotation Version and Release Date: PANTHER version 10.0 Released 2015-05-15

Analyzed List: Client Text Box Input (Homo sapiens) Change

Reference List: Homo sapiens (all genes in database) Change

Annotation Data Set: PANTHER GO-Slim Molecular Function

☑ Use the Bonferroni correction for multiple testing ?

Results ?

	Reference list	Client Text Box Input
Mapped IDs:	20814	400
Unmapped IDs:	0	106

Export results View: -- Please select a chart to display --

Displaying only results with P<0.05; click here to display all results

PANTHER GO-Slim Molecular Function	Homo sapiens (REF) #	Client Text Box Input #	expected	Fold Enrichment	+/-	P value
antioxidant activity (GO:0016209)	30	8	.58	> 5	+	2.89E-05
peroxidase activity (GO:0004601)	27	7	.52	> 5	+	2.06E-04
translation elongation factor activity (GO:0003746)	46	7	.88	> 5	+	6.30E-03
extracellular matrix structural constituent (GO:0005201)	76	9	1.46	> 5	+	3.58E-03
actin binding (GO:0003779)	176	20	3.38	> 5	+	7.71E-08
isomerase activity (GO:0016853)	166	17	3.19	> 5	+	6.93E-06
cysteine-type peptidase activity (GO:0008234)	92	9	1.77	> 5	+	1.53E-02
structural constituent of cytoskeleton (GO:0005200)	663	59	12.74	4.63	+	4.30E-20
metallopeptidase activity (GO:0008237)	170	15	3.27	4.59	+	2.70E-04
cytoskeletal protein binding (GO:0008092)	251	21	4.82	4.35	+	5.53E-06
peptidase inhibitor activity (GO:0030414)	226	18	4.34	4.14	+	1.07E-04
structural molecule activity (GO:0005198)	1034	82	19.87	4.13	+	1.53E-25
serine-type peptidase activity (GO:0008236)	322	24	6.19	3.88	+	4.97E-06
peptidase activity (GO:0008233)	630	45	12.11	3.72	+	1.41E-11
oxidoreductase activity (GO:0016491)	626	40	12.03	3.32	+	1.03E-08
enzyme inhibitor activity (GO:0004857)	362	18	6.96	2.59	+	4.90E-02
hydrolase activity (GO:0016787)	2205	75	42.38	1.77	+	1.39E-04
protein binding (GO:0005515)	2819	87	54.18	1.61	+	8.24E-04
catalytic activity (GO:0003824)	5209	140	100.11	1.40	+	9.51E-04
Unclassified	10020	118	192.56	.61	-	0.00E00
DNA binding (GO:0003677)	1878	12	36.09	.33	-	2.43E-04
kinase activity (GO:0016301)	778	3	14.95	.20	-	3.08E-02
sequence-specific DNA binding transcription factor activity (GO:0003700)	1462	4	28.10	< 0.2	-	1.48E-06

nucleic acid binding transcription factor activity (GO:0001071)	1646	4	31.63	< 0.2	-	5.32E-08

© Copyright 2016 Paul Thomas All Rights Reserved.
