# Supplementary material for: Unbiased analysis of senescence associated secretory phenotype (SASP) to identify common components following different genotoxic stresses
Source: Aging (Albany NY). 2016 Jun 9;8(7):1316–27. doi: 10.18632/aging.100971 (PMC4993333; doi:10.18632/aging.100971)
Supplement: Supplementary file 6 [file aging-08-1316-s006.pdf]

COMMON CANONICAL PATHS PRESENT IN SENESCENT A MSC and BM MSC

138 pathways

- Triacylglycerol Degradation
- fMLP Signaling in Neutrophils
- Signaling by Rho Family GTPases
- Hepatic Fibrosis / Hepatic Stellate Cell Activation
- Role of Tissue Factor in Cancer
- Ephrin Receptor Signaling
- Aspartate Degradation II
- Pyruvate Fermentation to Lactate
- Unfolded protein response
- Diphthamide Biosynthesis
- Uracil Degradation II (Reductive)
- Glutathione Redox Reactions I
- Leukocyte Extravasation Signaling
- IL-6 Signaling
- Caveolar-mediated Endocytosis Signaling
- Remodeling of Epithelial Adherens Junctions
- mTOR Signaling
- Telomerase Signaling
- Neuregulin Signaling
- Ephrin B Signaling
- Pentose Phosphate Pathway (Oxidative Branch)
- Sucrose Degradation V (Mammalian)
- RAN Signaling
- Guanosine Nucleotides Degradation III
- Hypoxia Signaling in the Cardiovascular System
- HIF1α Signaling
- Docosahexaenoic Acid (DHA) Signaling
- Rapoport-Luebering Glycolytic Shunt
- Prostate Cancer Signaling

- NAD Salvage Pathway II
- CD28 Signaling in T Helper Cells
- Death Receptor Signaling
- Glutathione-mediated Detoxification
- Parkinson's Signaling
- Granulocyte Adhesion and Diapedesis
- RhoGDI Signaling
- PCP pathway
- Adenosine Nucleotides Degradation II
- Phospholipases
- Thymine Degradation
- Tight Junction Signaling
- Colanic Acid Building Blocks Biosynthesis
- Huntington's Disease Signaling
- eNOS Signaling
- Pyrimidine Ribonucleotides Interconversion
- PPARα/RXRα Activation
- Agrin Interactions at Neuromuscular Junction
- Ceramide Signaling
- Bladder Cancer Signaling
- VEGF Signaling
- Cellular Effects of Sildenafil (Viagra)
- Urate Biosynthesis/Inosine 5'-phosphate Degradation
- Virus Entry via Endocytic Pathways
- Thyroid Hormone Biosynthesis
- p70S6K Signaling
- Ingenuity Canonical Pathways
- Regulation of Actin-based Motility by Rho
- ERK/MAPK Signaling
- Mitochondrial Dysfunction
- Nitric Oxide Signaling in the Cardiovascular System
- Oncostatin M Signaling
- Aryl Hydrocarbon Receptor Signaling

- Aldosterone Signaling in Epithelial Cells
- Maturity Onset Diabetes of Young (MODY) Signaling
- phagosome maturation
- Protein Ubiquitination Pathway
- p53 Signaling
- Complement System
- Agranulocyte Adhesion and Diapedesis
- phagosome formation
- Integrin Signaling
- PI3K/AKT Signaling
- Glioma Invasiveness Signaling
- Inhibition of Matrix Metalloproteases
- Lipid Antigen Presentation by CD1
- Purine Nucleotides Degradation II (Aerobic)
- Axonal Guidance Signaling
- PPAR Signaling
- TGF-β Signaling
- Semaphorin Signaling in Neurons
- Epithelial Adherens Junction Signaling
- Cdc42 Signaling
- ERK5 Signaling
- Gap Junction Signaling
- Crosstalk between Dendritic Cells and Natural Killer Cells
- RhoA Signaling
- IL-8 Signaling
- ILK Signaling
- Paxillin Signaling
- Inhibition of Angiogenesis by TSP1
- Gluconeogenesis I
- Melatonin Signaling
- Glutathione Redox Reactions II
- NADH Repair
- TCA Cycle II (Eukaryotic)

- Clathrin-mediated Endocytosis Signaling
- γ-glutamyl Cycle
- Coagulation System
- Pentose Phosphate Pathway (Non-oxidative Branch)
- GPCR-Mediated Integrat. of Enteroendocr. Sign. Exemplified by an L Cell
- Cell Cycle: G2/M DNA Damage Checkpoint Regulation
- LXR/RXR Activation
- Endoplasmic Reticulum Stress Pathway
- Airway Pathology in Chronic Obstructive Pulmonary Disease
- Role of p14/p19ARF in Tumor Suppression
- Actin Nucleation by ARP-WASP Complex
- 14-3-3-mediated Signaling
- Calcium Signaling
- Glycolysis I
- Sertoli Cell-Sertoli Cell Junction Signaling
- Corticotropin Releasing Hormone Signaling
- Intrinsic Prothrombin Activation Pathway
- Extrinsic Prothrombin Activation Pathway
- Regulation of Cellular Mechanics by Calpain Protease
- Germ Cell-Sertoli Cell Junction Signaling
- Pentose Phosphate Pathway
- Protein Kinase A Signaling
- FXR/RXR Activation
- Breast Cancer Regulation by Stathmin1
- Rac Signaling
- Apoptosis Signaling
- Myc Mediated Apoptosis Signaling
- Actin Cytoskeleton Signaling
- HIPPO signaling
- Acute Phase Response Signaling
- Pyrimidine Deoxyribonucleotides De Novo Biosynthesis I
- Calcium Transport I

- Pyrimidine Ribonucleotides De Novo Biosynthesis
- FAK Signaling
- Androgen Signaling
- Fcγ Receptor-mediated Phagocytosis in Macrophages and Monocytes
- Antigen Presentation Pathway
- IGF-1 Signaling
- Mitotic Roles of Polo-Like Kinase
- Glucocorticoid Receptor Signaling
- Antioxidant Action of Vitamin C
- NRF2-mediated Oxidative Stress Response
- Telomere Extension by Telomerase
- Atherosclerosis Signaling
